# Assessing mangrove sensitivity to thermal variations through a satellite-based analysis on the southern São Paulo coast, Brazil

**DOI:** 10.1007/s10661-026-16005-6

**Published:** 2026-10-09

**Authors:** Jakeline Baratto, Paulo Miguel de Bodas Terassi, Nádia Gilma Beserra de Lima, Emerson Galvani

**Affiliations:** Department of Geography, University of São Paulo, São Paulo, São Paulo, 05508-000 Brazil

**Keywords:** Cananéia-Iguape estuary, ERA5 reanalysis, MODIS vegetation products, Canopy greenness, Leaf area index, Mann-Kendall test

## Abstract

Mangroves near subtropical range limits may benefit from fewer cold constraints, yet warmer conditions can also increase atmospheric water demand. These competing possibilities were examined in the Cananéia-Iguape Coastal System, southeastern Brazil, by combining ERA5 2 m air temperature with MODIS normalized difference vegetation index (NDVI), enhanced vegetation index (EVI), and leaf area index (LAI). Air-temperature trends were assessed for 1979–2019 with the Mann-Kendall test and Sen’s slope. Temperature-vegetation associations were calculated for 2003–2019 from mangrove-wide means and from individual grid cells at annual, monthly, and fixed calendar-quarter scales. Mean annual air temperature increased by about 0.02 °C year⁻^1^; the highest quarter-scale rate occurred in July–September (0.03 °C year⁻^1^). Annual temperature correlated positively with NDVI (*r* = 0.61, *R*^2^ = 0.38, *p* = 0.009) and EVI (*r* = 0.55, *R*^2^ = 0.30, *p* = 0.022), whereas the LAI relationship was not significant (*r* = 0.27, *R*^2^ = 0.07, *p* = 0.304). The sign of the association changed within the year: NDVI and EVI were negative in July–September, while positive EVI and LAI relationships occurred in October-December. Significant grid-cell relationships occupied limited and discontinuous areas. The temperature record shows regional warming, whereas canopy associations vary among metrics, periods, and locations. Given the bivariate design and coarse ERA5 grid, these associations should not be interpreted as causal or as responses to temperature alone.

**Supplementary Information:**

The online version contains supplementary material available at https://doi.org/10.1007/s10661-026-16005-6.

## Introduction

Mangrove forests support fisheries, store carbon, trap sediment, and dissipate wave energy. Along the Brazilian coast, these functions help reduce exposure to erosion, storm surges, shoreline instability, and sediment loss while sustaining estuarine livelihoods (Giri et al., 2011; Tran et al., 2022). Their condition, however, depends on the interaction of climate with freshwater delivery, salinity, geomorphology, tidal inundation, and disturbance.

Temperature is especially relevant near poleward mangrove limits. Severe cold events can restrict recruitment and survival, whereas moderate warming may lengthen the period suitable for growth. The same warming can become stressful when it raises evaporative demand or coincides with weak freshwater input and high salinity (Cavanaugh et al., 2014, 2018; Osland et al., 2017a, b; Ward et al., 2016). A positive temperature response should therefore not be assumed across all seasons or stands (Alongi, 2015, 2022; Duke et al., 2017; Pastor-Guzman et al., 2018).

Air temperature and land surface temperature describe different physical quantities. Air temperature characterizes the atmosphere near 2 m above the surface, whereas land surface temperature is a radiometric estimate of the emitting land or canopy surface and is strongly affected by radiation, emissivity, and surface moisture. The variables may covary, but they cannot be used interchangeably (Guha & Govil, 2020; Yue et al., 2007). Here, thermal conditions are represented only by ERA5 2 m air temperature.

NDVI, EVI, and LAI also represent different canopy attributes. NDVI summarizes the red-near-infrared contrast and may saturate in dense vegetation. EVI reduces some atmospheric and background effects and often remains more responsive at high biomass. LAI is a retrieved structural variable that estimates one-sided leaf area per unit ground area. Differences among the three time series may thus reflect both ecological behavior and product-specific retrieval characteristics (Pastor-Guzman et al., 2018; Tran et al., 2022).

Previous work in the Cananéia-Iguape Coastal System (CICS) has addressed rainfall variability, vegetation-rainfall relationships, land surface temperature, and mangrove microclimate (Baratto et al., 2024a, b, 2025; Lima & Galvani, 2018). The remaining question was whether long-term atmospheric warming shows a consistent association with canopy metrics across the mapped mangrove domain and whether that association changes within the year. This question is pertinent at the subtropical margin of Brazilian mangroves, where seasonal temperature and freshwater variation can produce different constraints within one estuary.

Two objectives were addressed: first, to quantify temporal and spatial trends in ERA5 2 m air temperature over the CICS mangroves at annual, monthly, and fixed calendar-quarter scales during 1979–2019; second, to estimate associations between air temperature and NDVI, EVI, and LAI during their common 2003–2019 period using mangrove-wide and grid-cell calculations. Because the analysis is observational and bivariate, the reported correlations are interpreted as associations rather than evidence of an effect.

## Materials and methods

### Study area

**Fig. 1:**
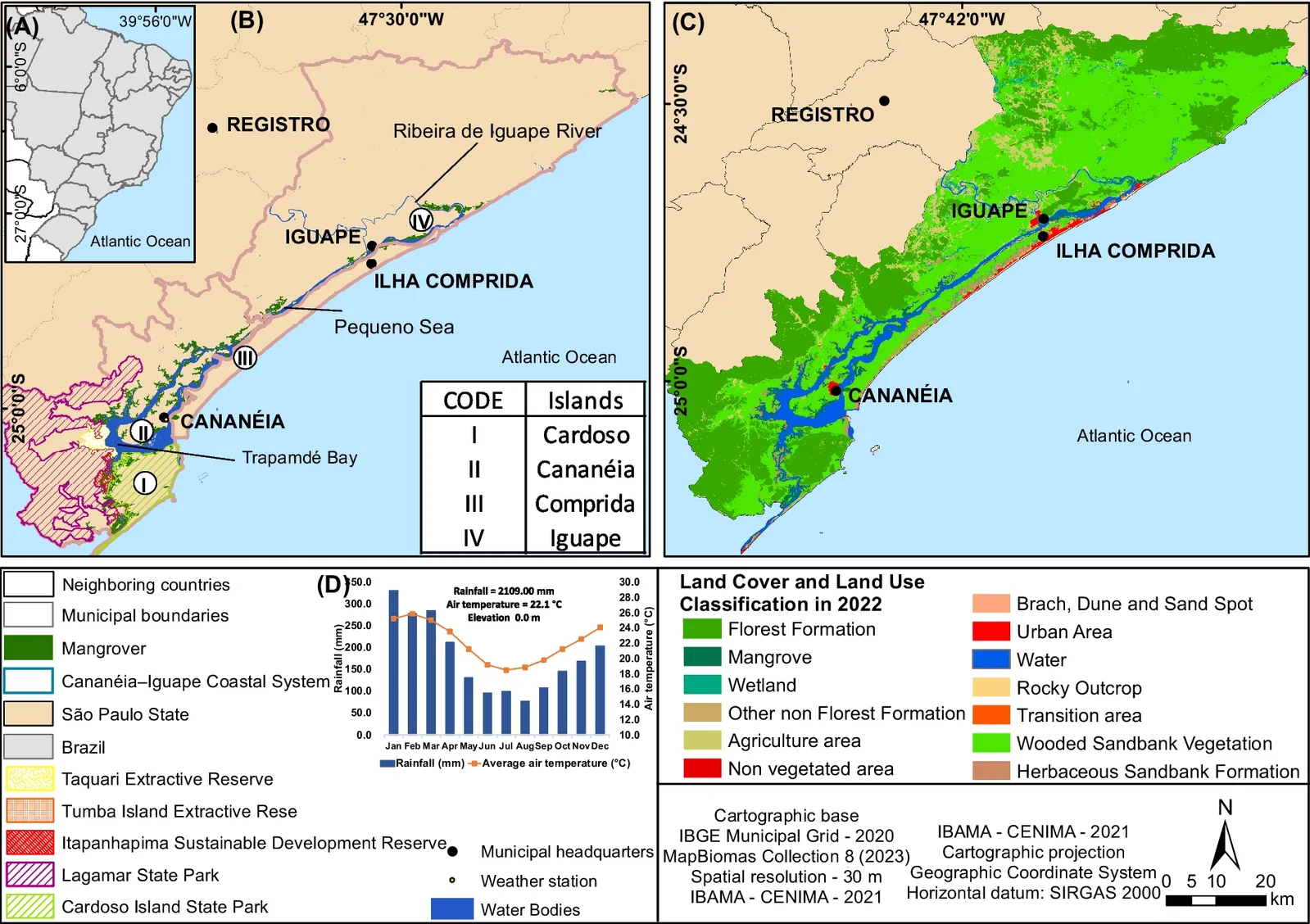
Location and environmental setting of the Cananéia-Iguape Coastal System (CICS), São Paulo, Brazil. **A** Regional setting. **B** Conservation units, municipal centers, and principal islands. **C** Land use and land cover in 2022. **D** Mean monthly precipitation and air temperature at Cananéia

The study area comprises the mangrove forests of the Cananéia-Iguape Coastal System on the southern coast of São Paulo State, within the municipalities of Cananéia, Iguape, and Ilha Comprida (Fig. 1). The system covers about 3414 km^2^, including approximately 125 km^2^ of mapped mangrove. Marine terraces, sandy barriers, tidal flats, estuarine channels, and fluvial-marine deposits create pronounced environmental gradients over relatively short distances (Ross, 2002; Souza & Perez Filho, 2019).

Atlantic Forest, restinga, wetlands, and mangroves occupy most of the CICS, while urban and agricultural land is more limited. Much of the mangrove area lies within state parks, extractive reserves, sustainable-development reserves, or the Juréia-Itatins Ecological Station. Protected status reduces direct clearing within these units but does not isolate the estuary from changes in surrounding catchments, which may alter runoff, sediment delivery, nutrients, and freshwater availability. Land-cover change was not included as a predictor, so the present analysis does not assign the mapped correlations to deforestation or conservation management (Giri et al., 2011; Ward et al., 2016).

The regional climate is humid subtropical with hot summers (Cfa; Alvares et al., 2013; Beck et al., 2018). At Cananéia, mean annual air temperature is about 22.1 °C and annual precipitation is approximately 2109 mm. Rainfall usually exceeds 200 mm month⁻^1^ from January through March and reaches its lowest values during much of June–August. This annual cycle provides the hydroclimatic setting for the temporal groupings used below.

### Satellite vegetation products

Mangrove-wide NDVI and EVI were obtained from MYD13Q1 Version 6.1, the Aqua MODIS vegetation-index product supplied as 16-day composites at 250 m resolution. Monthly and calendar-quarter grid-cell analyses of NDVI and EVI used MOD13Q1 Version 6.1, the corresponding Terra MODIS product with the same nominal resolution and compositing period. The 0.0001 scale factor was applied, and observations with SummaryQA = 0 were retained. LAI was obtained from MOD15A2H Version 6.1, a Terra MODIS 8-day composite at 500 m resolution (Didan, 2021a, b; Myneni et al., 2021). The common vegetation-temperature interval extends from January 2003 through December 2019. Google Earth Engine provided data access, quality filtering, temporal aggregation, mangrove masking, grid alignment, and export (Gorelick et al., 2017).

MODIS was chosen because its regular observations support a continuous multi-year record in a persistently cloudy coastal environment. The 250- and 500-m pixels cannot resolve every narrow fringe, channel margin, or ecotone, and mixed pixels remain possible. Monthly values were formed from quality-screened composites; annual values were calculated from the monthly series. For calendar-quarter analysis, monthly observations within each fixed 3-month group were pooled over 2003–2019, yielding no more than 51 complete pairs per group. No vegetation value was interpolated to replace a missing observation. Because the mangrove-wide and grid-cell analyses use Aqua and Terra vegetation-index products, respectively, their results are treated as complementary rather than interchangeable.

### ERA5 air temperature

Near-surface atmospheric temperature was represented by the ERA5 daily mean 2 m air temperature field produced by the European Centre for Medium-Range Weather Forecasts. ERA5 combines a forecast model with assimilated observations and is distributed on an atmospheric grid of approximately 0.25° (Hersbach et al., 2020; Soci et al., 2024). Temperatures were converted from kelvin to degrees Celsius before monthly, calendar-quarter, and annual means were calculated.

Temperature trends were evaluated for 1979–2019; correlations with the MODIS metrics used the overlapping 2003–2019 period. For the grid-cell analysis, ERA5 temperature and the 500 m LAI product were resampled to the 250 m vegetation-index grid by bicubic interpolation in Google Earth Engine. This alignment enabled cell-by-cell comparison while preserving the informational limits of ERA5’s native grid. The resulting maps portray relationships with a regional atmospheric field rather than measurements of stand-level mangrove microclimate.

### Temporal aggregation and analysis units

Two spatial units were analyzed. Mangrove-wide associations use the mean of all valid mangrove pixels at each time step. Grid-cell associations were calculated separately for each 250 m cell from the complete temperature-vegetation pairs available at that location. Accordingly, the annual points in the mangrove-wide ERA5 2 m air temperature results and the corresponding annual vegetation metrics are mean values across all valid mangrove pixels. Month-specific and calendar-quarter mangrove-wide analyses used complete temperature-vegetation pairs, while grid-cell analyses calculated coefficients separately for each 250 m cell.

The fixed 3-month groups were JFM (January–March), AMJ (April–June), JAS (July–September), and OND (October–December). They are calendar quarters, not the standard austral meteorological seasons DJF, MAM, JJA, and SON. These fixed calendar quarters were retained because they represent four distinct phases of the local annual cycle: peak summer warmth and rainfall, autumn cooling, the coolest and comparatively drier interval, and the spring-to-early-summer recovery of temperature and rainfall (Baratto et al., 2024b, 2025; Ferreira & Reboita, 2022). The text therefore uses the term calendar quarter rather than season.

### Pearson correlation and linear regression

Pearson’s product-moment coefficient was used to describe the direction and magnitude of the linear association between air temperature (*T*) and each vegetation metric (*V*) (Pearson, 1895). For *n* complete pairs, the coefficient was calculated with Eq. (1).

1 $$r=\frac{\sum_{i=1}^{n}\left({T}_{i}-\overline{T}\right)\left({V}_{i}-\overline{V}\right)}{\sqrt{\sum_{i=1}^{n}{\left({T}_{i}-\overline{T}\right)}^{2}\sum_{i=1}^{n}{\left({V}_{i}-\overline{V}\right)}^{2}}}$$

In Eq. (1), *T*ᵢ and *V*ᵢ are paired temperature and vegetation observations, *T̄* and *V̄* are their sample means, and *n* is the number of complete pairs. For an ordinary least-squares regression with one predictor and an intercept, the coefficient of determination is given by Eq. (2).

2 $${R}^{2}={r}^{2}$$

Only complete temperature-vegetation pairs were used; a time step was omitted if either value was unavailable. Scatterplots were reviewed for obvious curvature and influential observations before the coefficients were summarized. Formal tests of bivariate normality were not conducted, and neither prewhitening, correction for serial correlation, nor robust-regression diagnostics were applied. Accordingly, *r* and *R*^*2*^ are interpreted as descriptive measures of linear association, whereas the associated *p* values are considered nominal. The primary significance threshold was *p* ≤ 0.05. The 0.05 < *p* ≤ 0.10 class is retained in some supplementary maps solely for comparison with the original outputs and is interpreted as exploratory.

For cartographic display, *r* was classified as strong negative (−1.00 to −0.70), moderate negative (−0.69 to −0.40), weak negative or near zero (−0.39 to 0.00), weak positive (0.01 to 0.39), moderate positive (0.40 to 0.69), or strong positive (0.70 to 1.00). Identical limits were used at annual, monthly, and calendar-quarter scales to permit direct visual comparison. The grid-cell implementation follows the general pixel-wise approach described by Kumari et al. (2021). Online Resource 1 identifies the R procedures (R Core Team, 2024) used for mangrove-wide calculations and the Google Earth Engine procedures used for the spatial analyses.

### Mann–Kendall trend test and Sen’s slope

Trend tests were applied only to ERA5 air temperature. NDVI, EVI, and LAI were not subjected to Mann-Kendall trend analysis. For a series x₁, …, xₙ, the Mann-Kendall statistic *S* was calculated from all ordered observation pairs (Kendall & Gibbons, 1990; Sneyers, 1975), as specified in Eq. (3).

3 $$S=\sum\nolimits_{i=1}^{n-1}\sum\nolimits_{j=i+1}^{n}\mathrm{s}\mathrm{g}\mathrm{n}\left({x}_{j}-{x}_{i}\right)$$

In Eq. (3), sgn(*x*ⱼ−*x*ᵢ) takes the value 1, 0, or −1 when the difference is positive, zero, or negative. The test used a two-sided alternative. Trend magnitude was estimated with Sen’s median pairwise slope (Sen, 1968), defined in Eq. (4).

4 $$\widehat{\beta }={\mathrm{m}\mathrm{e}\mathrm{d}\mathrm{i}\mathrm{a}\mathrm{n}}_{1\le i<j\le n}\left(\frac{{x}_{j}-{x}_{i}}{j-i}\right)$$

In Eq. (4),* β̂* is the median of the slopes between observations *x*ᵢ and *x*ⱼ recorded at ordered times *i* and *j*, where *i* < *j*. Slopes are reported in °C year⁻^1^ and, when useful for interpretation, °C decade⁻^1^. The primary threshold was *p* ≤ 0.05; the 0.05 < *p* ≤ 0.10 category is retained only to document the original spatial workflow. No prewhitening, variance correction for serial correlation, false-discovery-rate procedure, or field-significance test was applied.

## Results

### Air-temperature climatology

**Fig. 2:**
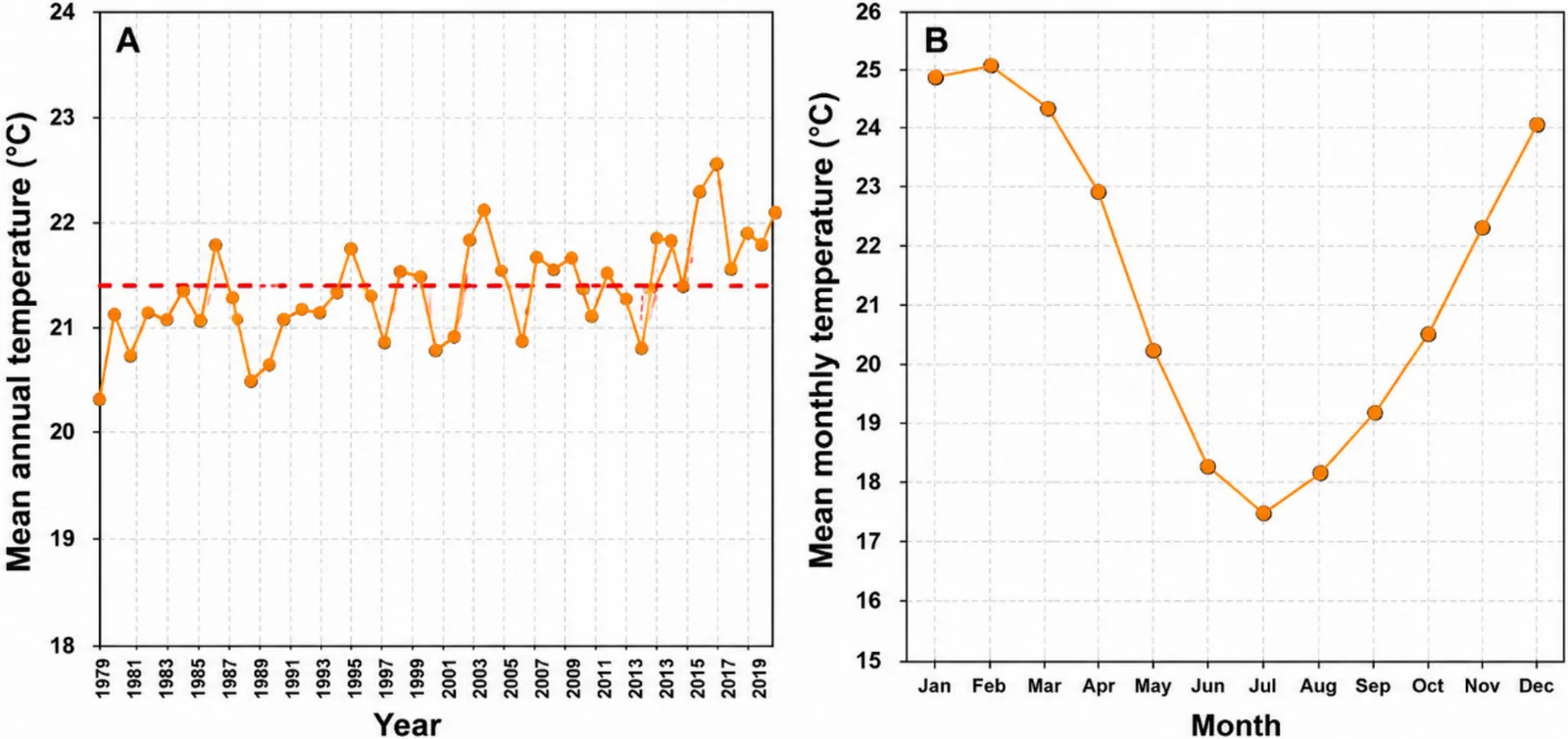
Area-mean ERA5 2 m air temperature over the CICS during 1979–2019. **A** Annual means and **B** monthly climatological means

The 1979–2019 area-mean ERA5 temperature was 21.5 °C. Annual means ranged from 20.6 °C in 1979 to 22.6 °C in 2015, with a variance of 0.19°C^2^ and a standard deviation of 0.43 °C. The monthly climatology ranged from 17.5 °C in July to 25.2 °C in February (Fig. 2).

**Fig. 3:**
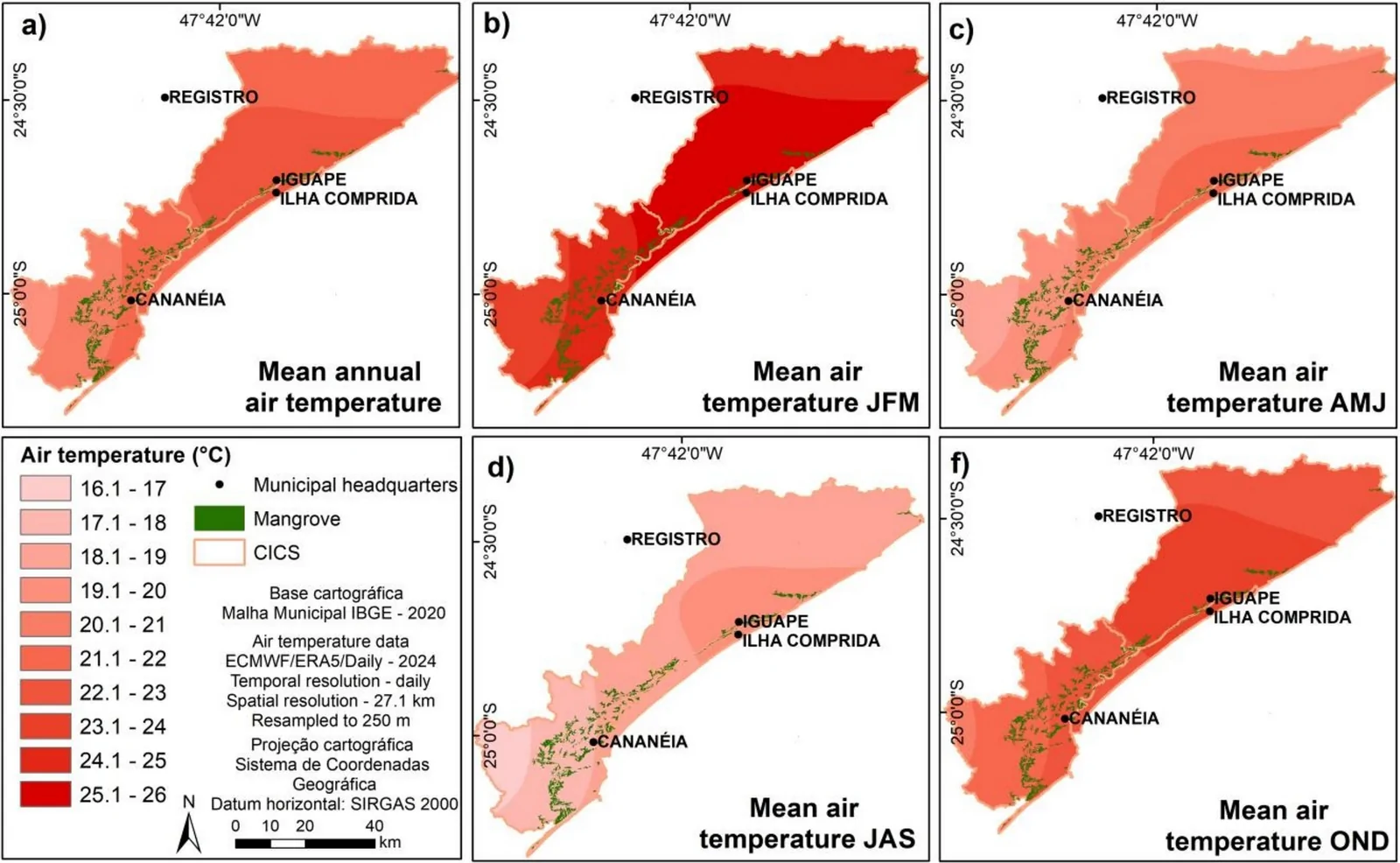
Spatial distribution of mean annual and seasonal air temperature (°C) in the Cananéia–Iguape Coastal System (CICS) from 1979 to 2020. Panels represent **a** annual mean, **b** JFM (January–February–March), **c** AMJ (April–May–June), **d** JAS (July–August–September), **e** map legend, and f OND (October–November–December). Colors indicate temperature ranges, green areas represent mangrove forests, and black dots mark municipal seats

Across the mapped domain, annual mean temperature ranged from about 19.0 °C in the western sector to 22.5 °C in the north-central CICS. JFM had the highest quarter mean (approximately 23.1–26.0 °C), and JAS had the lowest (approximately 16.1–19.0 °C). The central sector was comparatively warm in each calendar quarter (Fig. 3).

### Temporal and spatial trends in air temperature

**Fig. 4:**
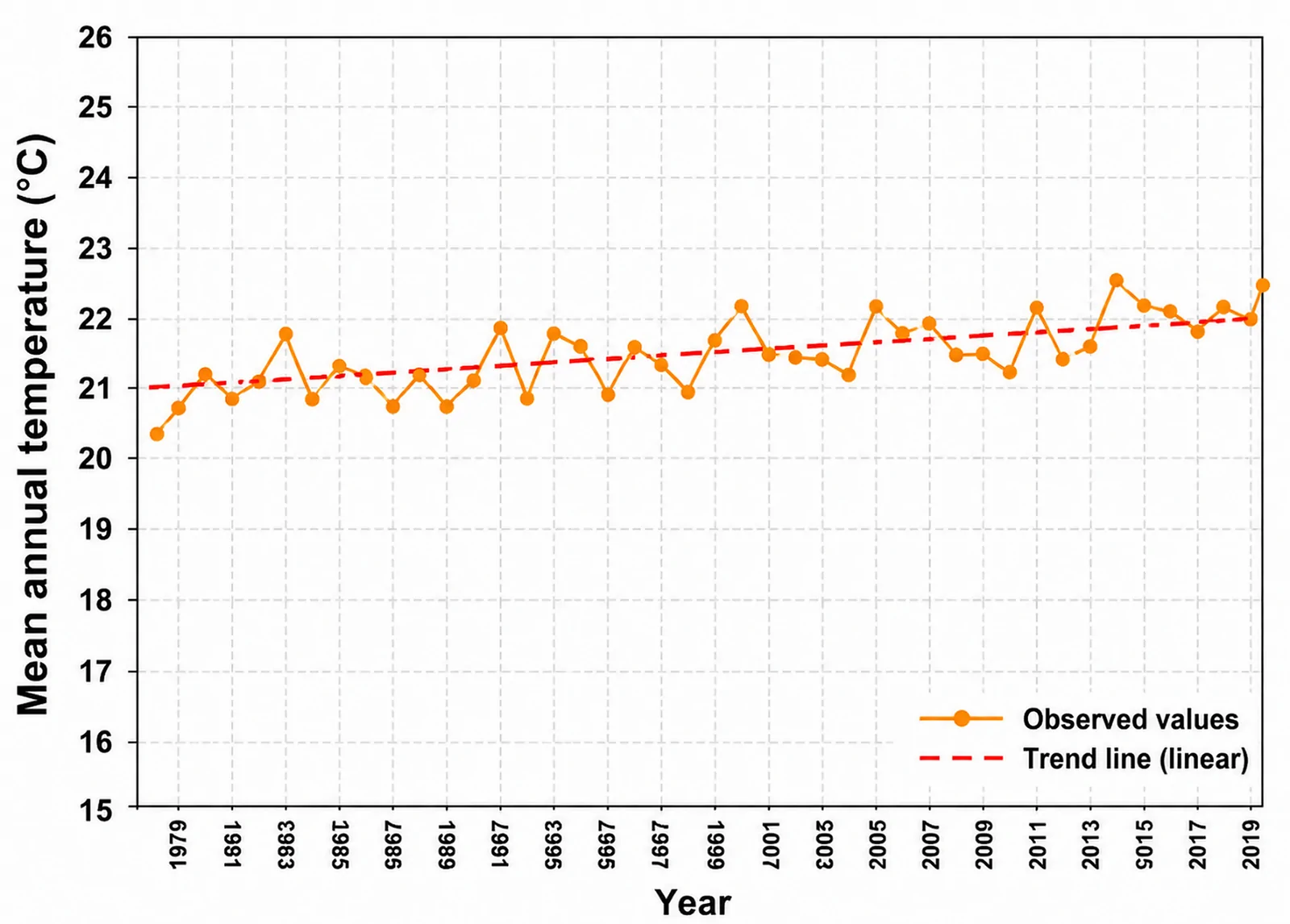
Annual area-mean ERA5 2 m air temperature over the CICS mangrove domain during 1979–2019. The dashed ordinary least-squares line is included for visualization; statistical inference is based on the Mann–Kendall test and Sen’s slope

Annual area-mean temperature increased during 1979–2019 (*p* < 0.01). Sen’s slope was approximately 0.02 °C year⁻^1^, or 0.20 °C decade⁻^1^ (Fig. 4). Annual grid-cell slopes were mainly positive, but none met *p* ≤ 0.05 (Fig. S1 in Online Resource 2).

**Fig. 5:**
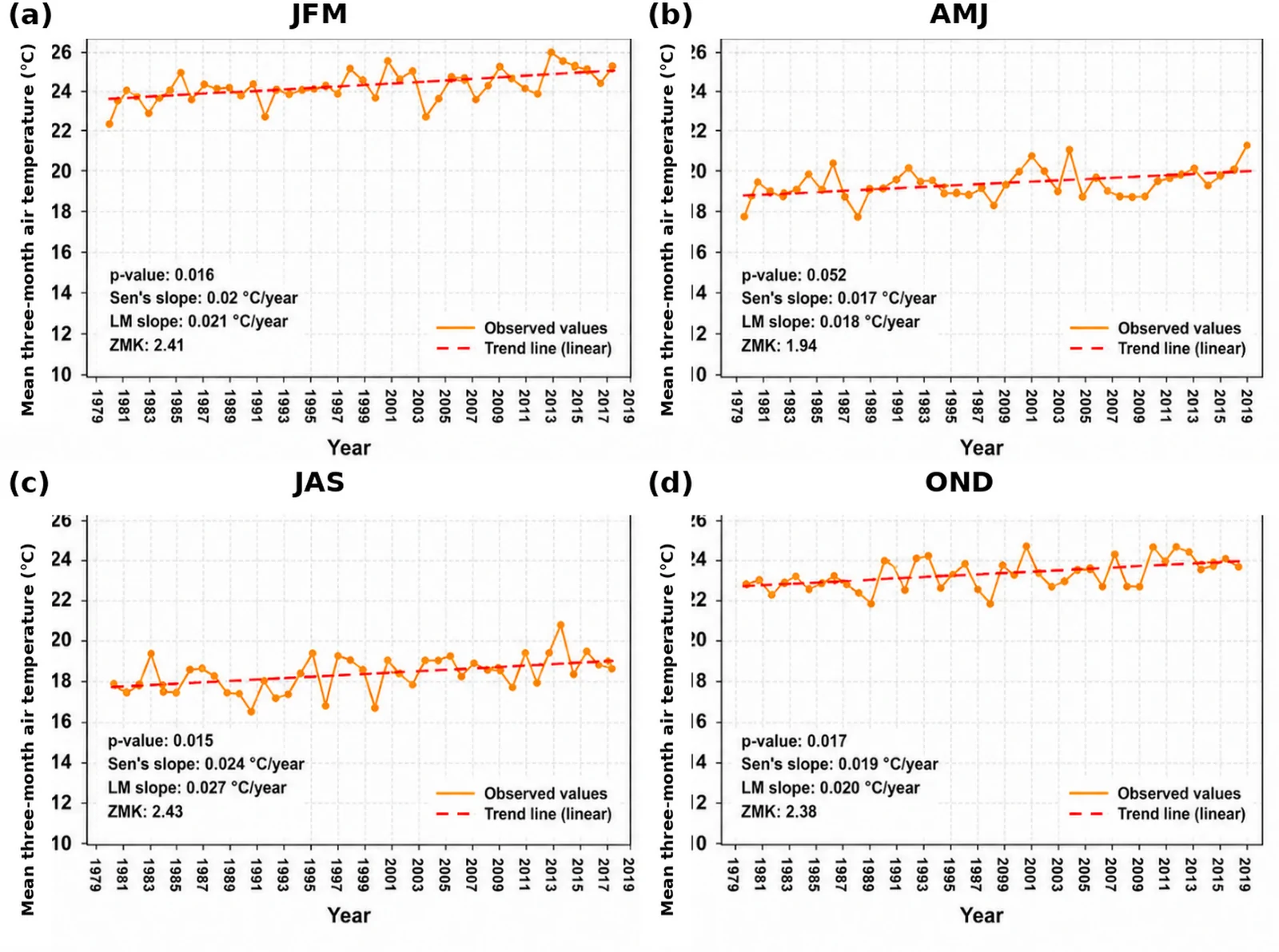
Temporal trends in mean seasonal temperature for the mangrove areas of the Cananéia–Iguape Coastal System (CICS) from 1979 to 2019. Seasonal periods are represented by **a** JFM (January–February–March), **b** AMJ (April–May–June), **c** JAS (July–August–September), and **d** OND (October–November–December)

Area-mean slopes were positive in all calendar quarters. JAS had the largest estimate, about 0.03 °C year⁻^1^ (*p* < 0.01). JFM and OND increased by about 0.02 °C year⁻^1^ (*p* < 0.01). AMJ had a comparable slope, although *p* = 0.052 did not meet the primary threshold (Fig. 5).

**Fig. 6:**
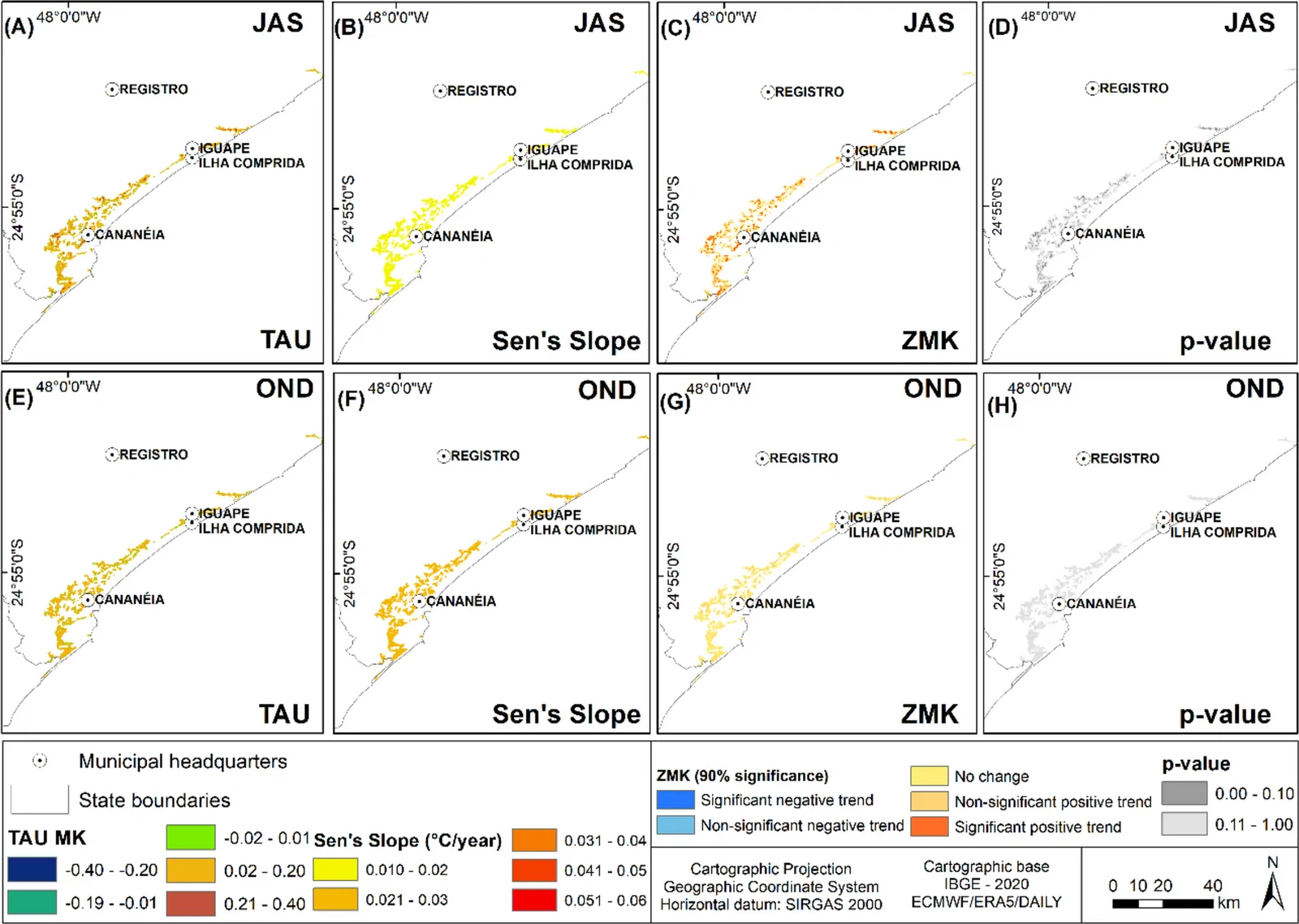
Spatial trends in mean seasonal (JAS and OND) temperature for the mangrove areas of the Cananéia–Iguape Coastal System (CICS) for JAS (July–August–September) and OND (October–November–December) from 1979 to 2019. The analysis includes **A**, **E** Kendall’s Tau (*t*), **B**, **F** Sen’s Slope estimator (°C/year), **C**,** G** Mann–Kendall test (ZMK) at 90% significance, and **D**, **H** statistical significance (*p*-value)

During JAS, positive grid-cell trends meeting *p* ≤ 0.05 covered about 19.5% of the mangrove domain. These cells occurred mainly along the continental margin, near the Ribeira de Iguape River mouth, and south of Cananéia; most slopes were 0.02–0.03 °C year⁻^1^. OND slopes were also predominantly positive, but few cells met *p* ≤ 0.05 (Fig. 6). JFM and AMJ maps are provided in Fig. S2.

**Fig. 7:**
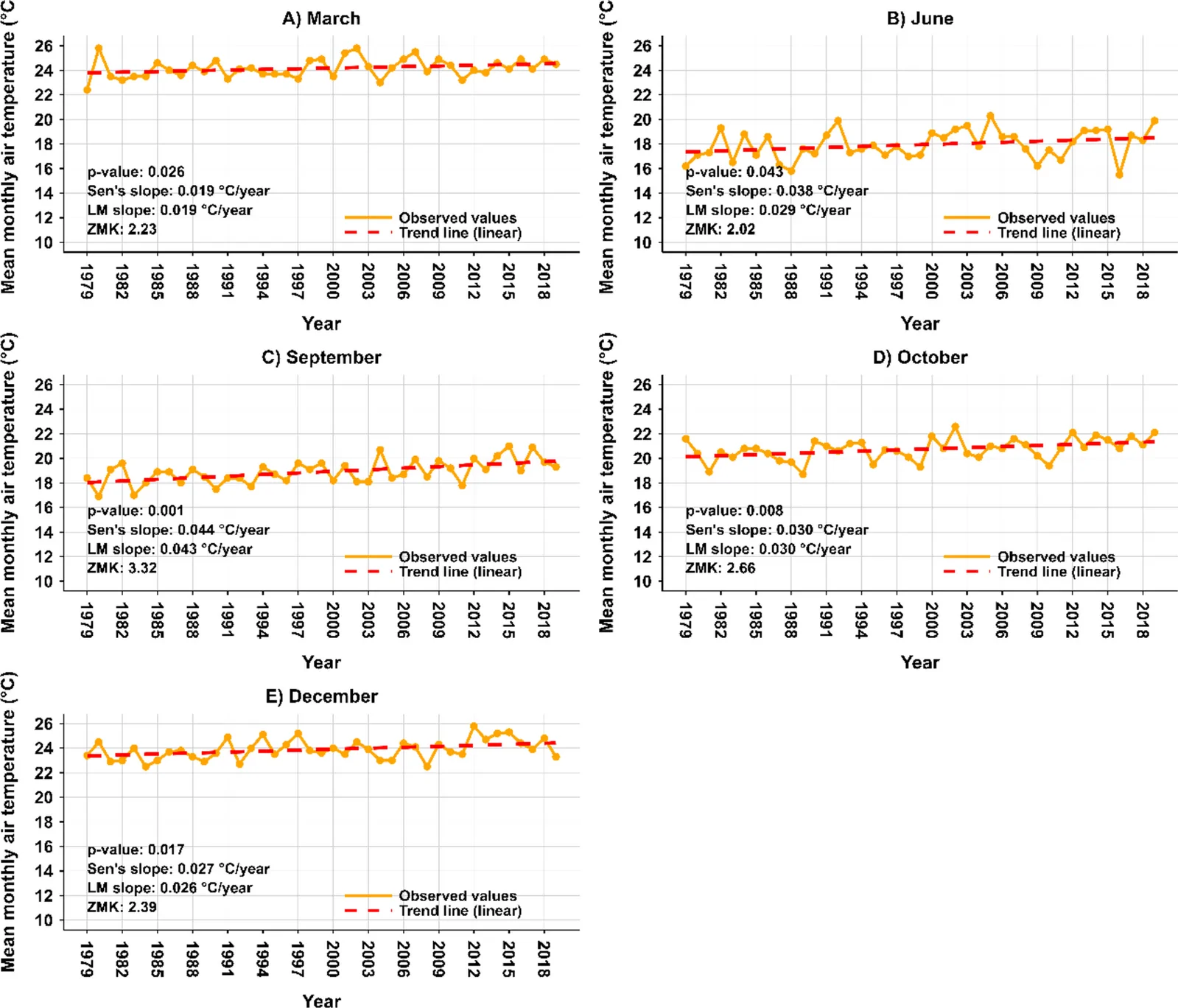
Monthly area-mean ERA5 2 m air temperature trends that met *p* ≤ 0.05 during 1979–2019: **A** March, **B** June, **C** September, **D** October, and **E** December

All monthly area-mean slopes were positive. March, June, September, October, and December met *p* ≤ 0.05. September and June had the largest estimates, approximately 0.04 °C year⁻^1^, followed by October and December at about 0.03 °C year⁻^1^ (Fig. 7).

**Fig. 8:**
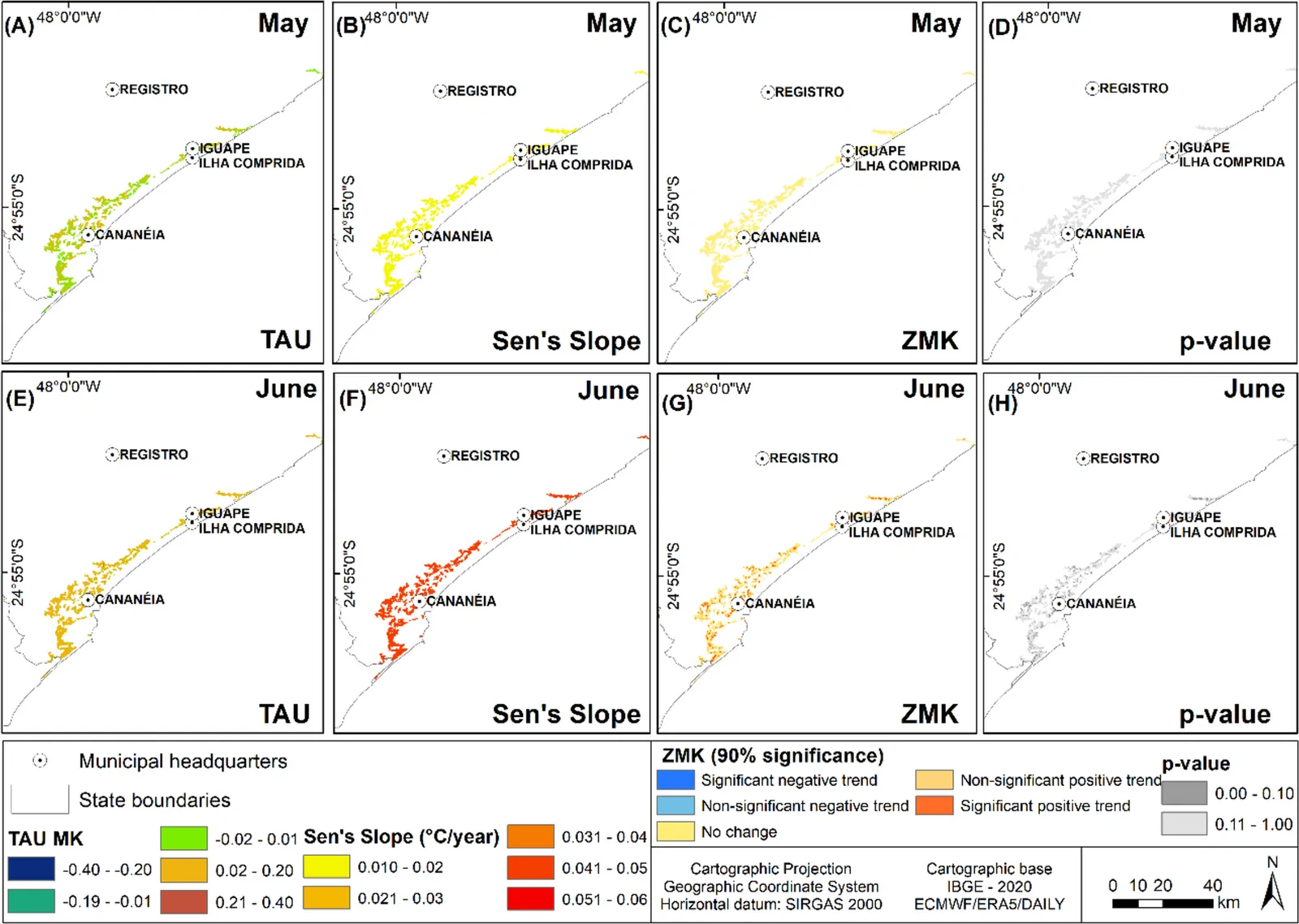
Spatial trends in mean monthly temperature for May and June in the Cananéia–Iguape Coastal System (CICS) for May and June from 1979 to 2019. The analysis includes **A**, **E** Kendall’s Tau (*t*), **B**, **F** Sen’s Slope estimator (°C/year), **C**, **G** Mann-Kendall test (ZMK) at 90% significance, and **D**, **H** statistical significance (*p*-value)

In June, grid cells meeting *p* ≤ 0.05 occupied about 16% of the mangrove domain. The largest slopes, 0.05–0.06 °C year⁻^1^, occurred near Papagaios Island and in northern Cananéia. May slopes were mainly nonsignificant (Fig. 8).

**Fig. 9:**
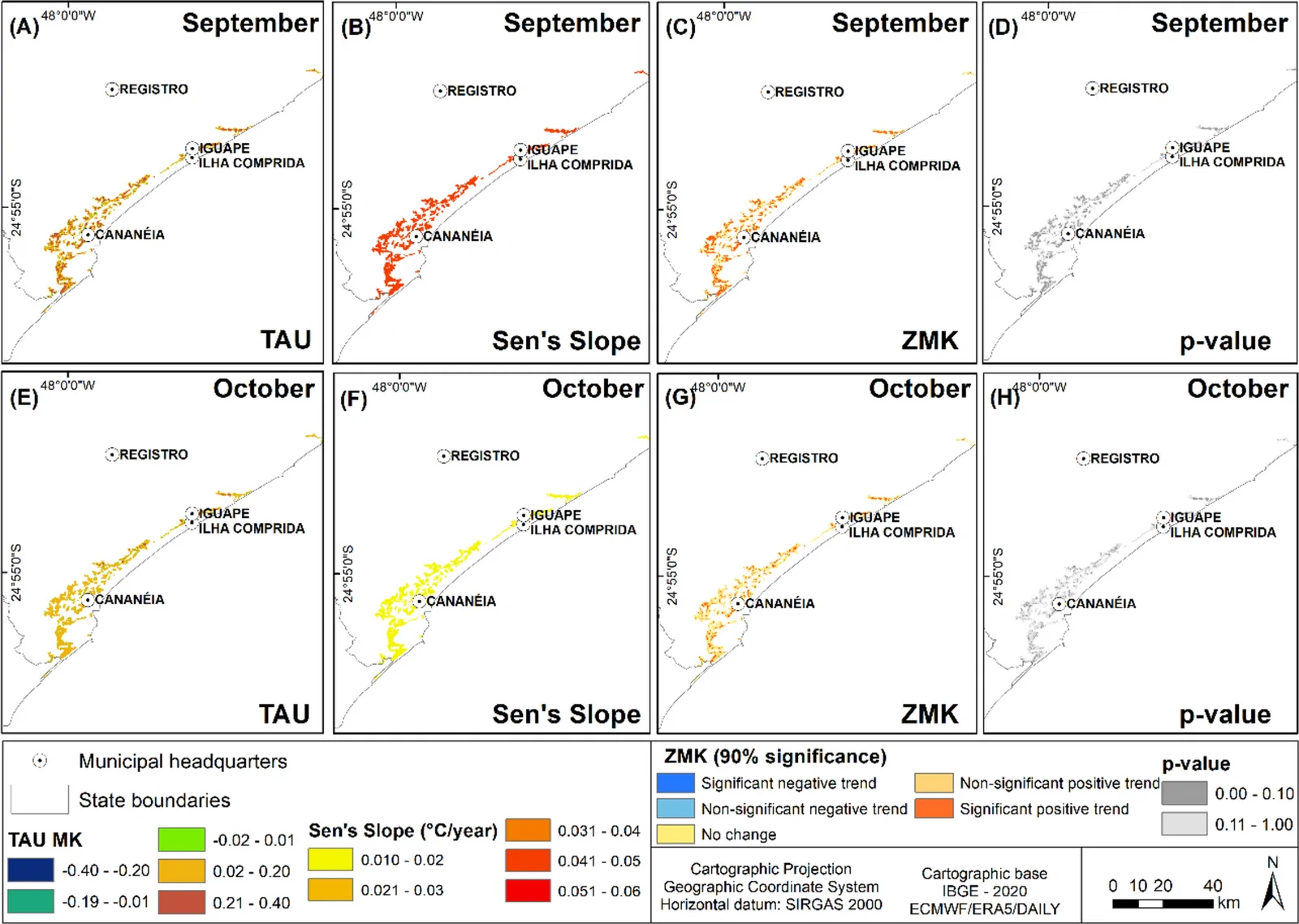
Spatial trends in mean monthly temperature for September and October in the Cananéia–Iguape Coastal System (CICS) for September and October from 1979 to 2019. The analysis includes **A**, **E** Kendall’s Tau (*t*), **B**, **F** Sen’s Slope estimator (°C/year), **C**, **G** Mann-Kendall test (ZMK) at 90% significance, and **D**, **H** statistical significance (*p*-value)

September had the greatest monthly coverage of warming at *p* ≤ 0.05, about 39% of the mangrove domain. Slopes of 0.05–0.06 °C year⁻^1^ were common in northern and southern Cananéia. October had a smaller significant area (approximately 17.5%) and slopes near 0.02 °C year⁻^1^ (Fig. 9). In December, significant warming was confined to roughly 5% of the domain, principally in northern and central sectors (Fig. S6). Maps for January–February, March–April, and July–August appear in Figs. S3-S5.

### Annual associations between air temperature and vegetation indices

**Fig. 10:**
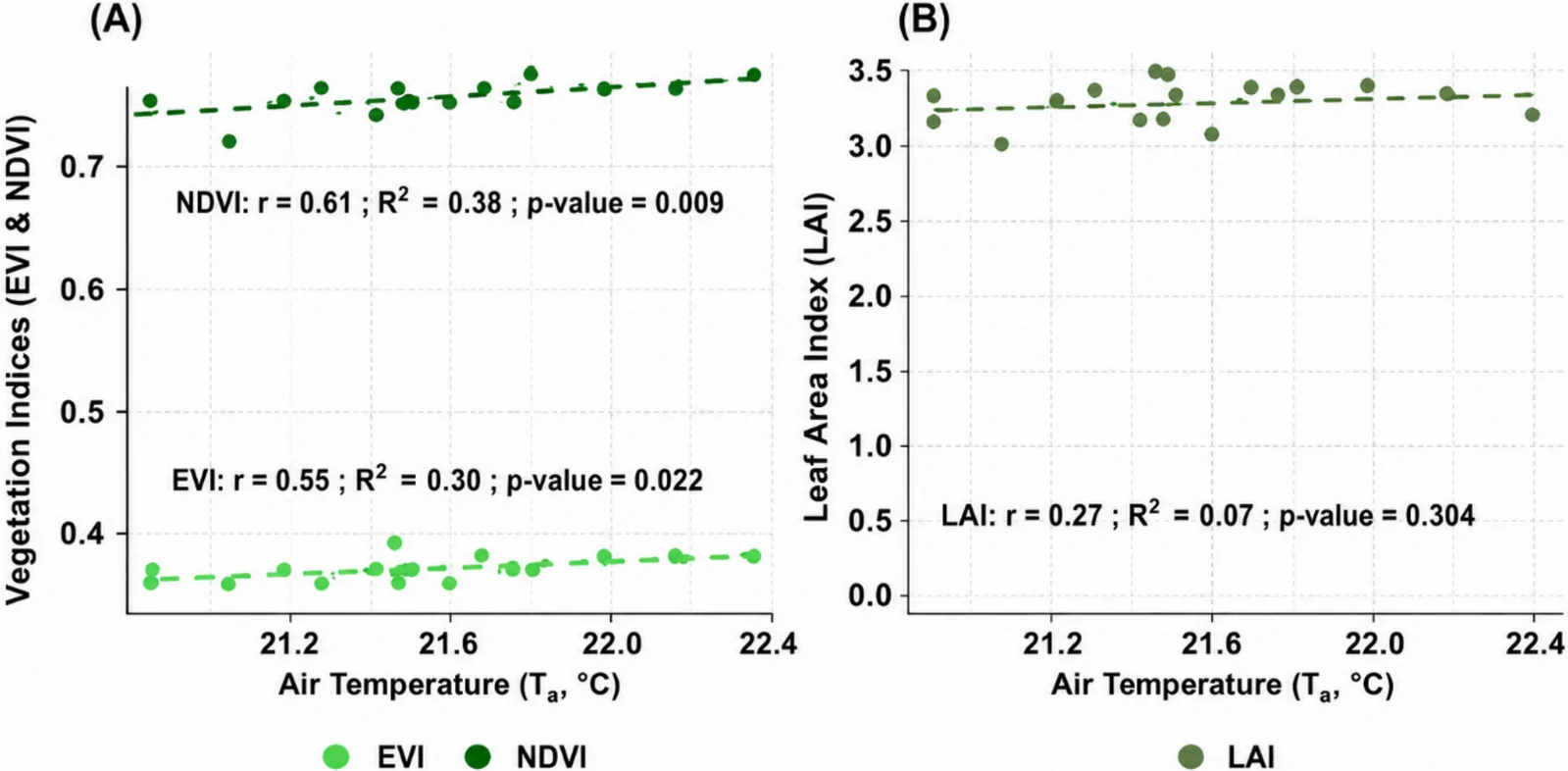
Linear regression between annual mean air temperature (T*a*) and mangrove vegetation indices (NDVI, EVI, and LAI) in the Cananéia–Iguape Coastal System (CICS). **A** Relationship for NDVI and EVI. **B** Relationship for LAI. The plots include the Pearson correlation coefficient (*r*), the coefficient of determination (*R*^2^), and the statistical significance (*p*-value). Each annual point represents the mean of all valid mangrove pixels for the respective year

Annual mangrove-wide temperature correlated positively with NDVI (*r* = 0.61, *R*^2^ = 0.38, *p* = 0.009) and EVI (*r* = 0.55, *R*^2^ = 0.30, *p* = 0.022). The LAI coefficient was smaller and not significant (*r* = 0.27, *R*^2^ = 0.07, *p* = 0.304; Fig. 10).

**Fig. 11:**
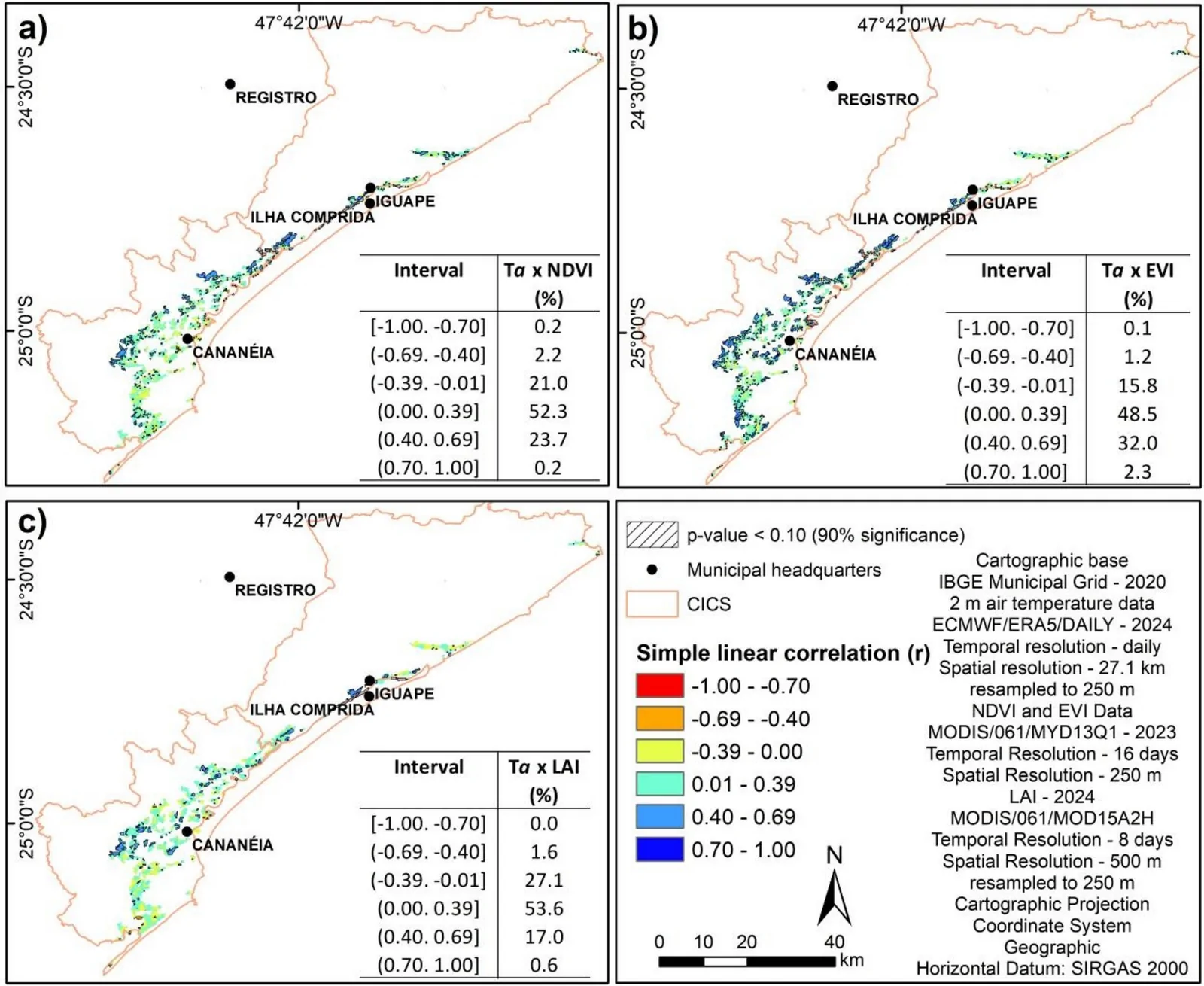
Annual grid-cell Pearson correlations between ERA5 2 m air temperature and **a** NDVI, **b** EVI, and **c** LAI during 2003–2019. Hatching marks the exploratory *p* ≤ 0.10 class

Annual grid-cell coefficients varied in sign and magnitude, and only a small part of the mapped area met *p* ≤ 0.05 (Fig. S7). Moderate positive EVI coefficients occupied about 32.0% of the domain and strong positive coefficients 2.3%. Strong positive coefficients occupied about 0.7% for NDVI and 0.6% for LAI. Positive NDVI and EVI values were concentrated south of Iguape and in central Cananéia; negative coefficients occurred in parts of Cananéia and southern Ilha do Cardoso (Fig. 11).

### Monthly associations between air temperature and vegetation indices

**Fig. 12:**
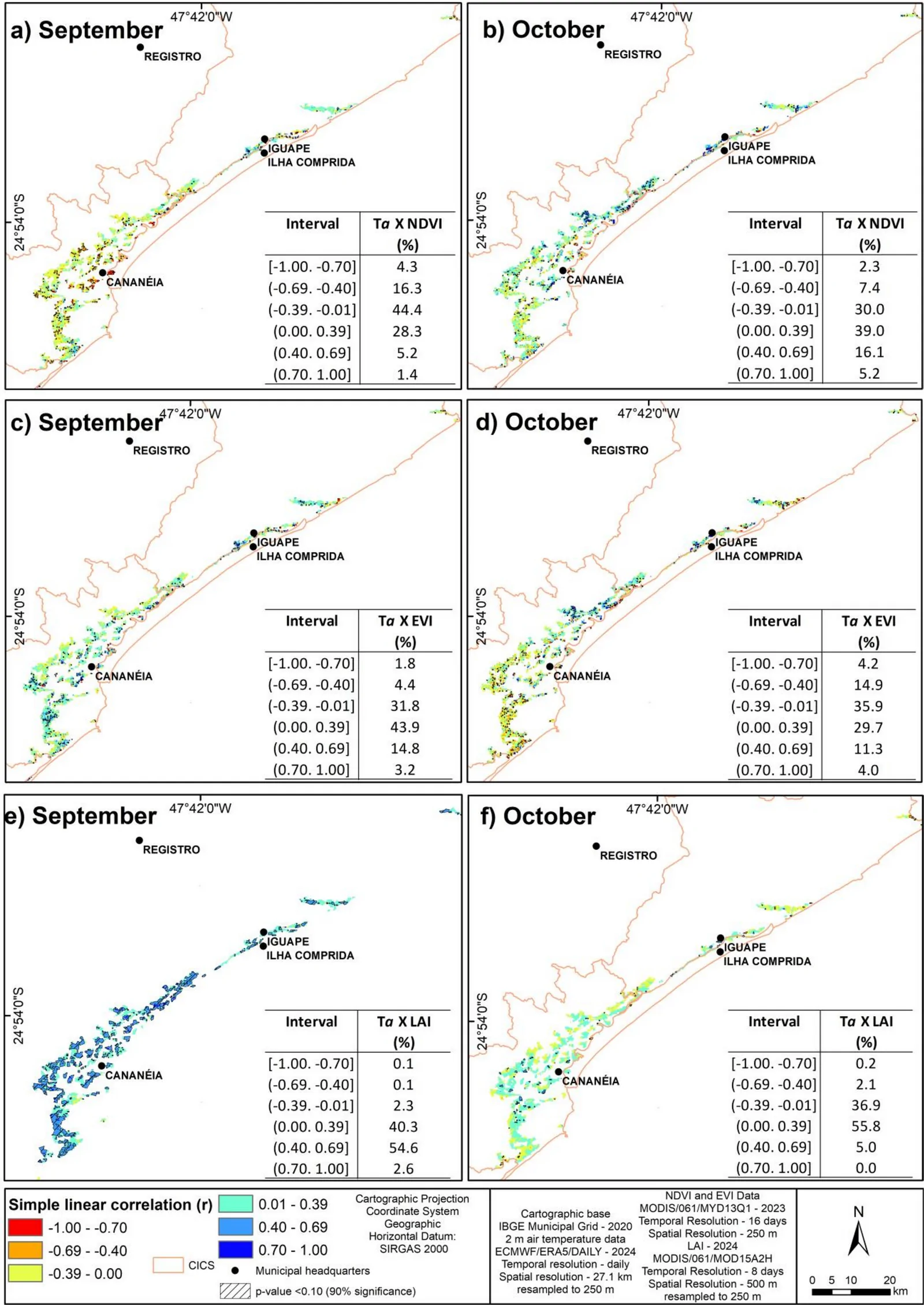
Monthly grid-cell correlations for September and October during 2003–2019. **a**, **b** NDVI. **c**, **d** EVI. **e**, **f** LAI. September is in the left column and October in the right column

**Fig. 13:**
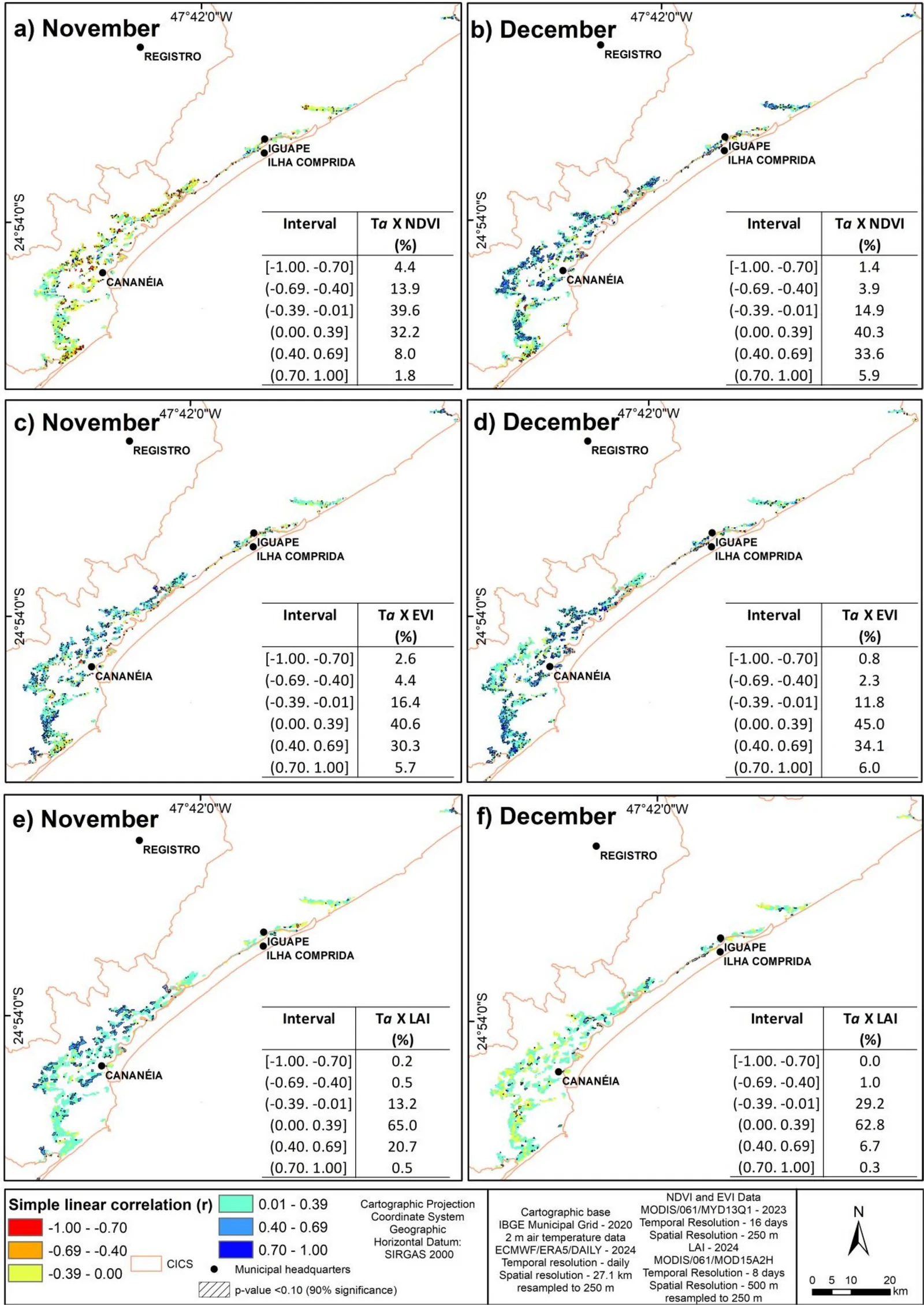
Monthly grid-cell correlations for November and December during 2003–2019. **a**, **b** NDVI. **c**, **d** EVI. **e**, **f** LAI. November is in the left column and December in the right column

Month-specific mangrove-wide coefficients differed among indices (Table 1). NDVI was positive in August and December (*r* = 0.65 in both months). LAI was positive in January (*r* = 0.64), February (*r* = 0.52), September (*r* = 0.66) (Fig. 12), and November (*r* = 0.48) (Fig. 13). No monthly EVI coefficient met *p* ≤ 0.05.

**Table 1:** Pearson correlation coefficient (*r*), coefficient of determination (*R*^2^), and nominal *p* value for monthly area-mean associations between ERA5 2 m air temperature and vegetation indices during 2003–2019

Month	NDVI	EVI	LAI
**Jan**	*r* = 0.17	*r* = 0.00	***r* = 0.64**
	*R*^2^ = 0.03	*R*^2^ = 0.00	***R***^**2**^** = 0.41**
	*p* = 0.52	*p* = 0.99	***p* = 0.01**
**Feb**	*r* = − 0.01	*r* = 0.31	***r* = 0.52**
	*R*^2^ = 0.00	*R*^2^ = 0.09	***R***^**2**^** = 0.27**
	*p* = 0.98	*p* = 0.23	***p* = 0.03**
**Mar**	*r* = 0.33	*r* = − 0.11	*r* = 0.32
	*R*^2^ = 0.11	*R*^2^ = 0.01	*R*^2^ = 0.10
	*p* = 0.19	*p* = 0.68	*p* = 0.21
**Apr**	*r* = 0.20	*r* = 0.22	*r* = − 0.04
	*R*^2^ = 0.04	*R*^2^ = 0.05	*R*^2^ = 0.00
	*p* = 0.44	*p* = 0.39	*p* = 0.88
**May**	*r* = 0.29	*r* = 0.17	*r* = − 0.23
	*R*^2^ = 0.09	*R*^2^ = 0.03	*R*^2^ = 0.05
	*p* = 0.25	*p* = 0.52	*p* = 0.37
**Jun**	*r* = 0.24	*r* = 0.26	*r* = 0.13
	*R*^2^ = 0.06	*R*^2^ = 0.07	*R*^2^ = 0.02
	*p* = 0.36	*p* = 0.32	*p* = 0.63
**Jul**	*r* = 0.08	*r* = − 0.26	*r* = 0.18
	*R*^2^ = 0.01	*R*^2^ = 0.07	*R*^2^ = 0.03
	*p* = 0.76	*p* = 0.32	*p* = 0.50
**Aug**	***r* = 0.65**	*r* = − 0.09	*r* = 0.24
	***R***^**2**^** = 0.42**	*R*^2^ = 0.01	*R*^2^ = 0.06
	***p* < 0.005**	*p* = 0.74	*p* = 0.36
**Sep**	*r* = 0.02	*r* = − 0.09	***r* = 0.66**
	*R*^2^ = 0.00	*R*^2^ = 0.01	***R***^**2**^** = 0.43**
	*p* = 0.95	*p* = 0.73	***p* < 0.005**
**Oct**	*r* = 0.08	*r* = − 0.14	*r* = − 0.01
	*R*^2^ = 0.01	*R*^2^ = 0.02	*R*^2^ = 0.00
	*p* = 0.79	*p* = 0.62	*p* = 0.97
**Nov**	*r* = 0.12	*r* = 0.32	***r* = 0.48**
	*R*^2^ = 0.02	*R*^2^ = 0.10	***R***^**2**^** = 0.23**
	*p* = 0.64	*p* = 0.21	***p* = 0.05**
**Dec**	***r* = 0.65**	*r* = 0.34	*r* = 0.15
	***R***^**2**^** = 0.43**	*R*^2^ = 0.12	*R*^2^ = 0.02
	***p* < 0.005**	*p* = 0.18	*p* = 0.57

Values of r and R² were rounded independently from full-precision output; small differences may therefore arise when the displayed r is squared. Values reported as *p* = 0.00 in the source output are shown as *p* 0.005. Bold values indicate statistically significant Pearson correlations (nominal *p* ≤ 0.05) and their associated r and R² values. P values were not adjusted for temporal autocorrelation or multiple testing Values of r and R² were rounded independently from full-precision output; small differences may therefore arise when the displayed r is squared. Values reported as *p* = 0.00 in the source output are shown as *p* 0.005. Bold values indicate statistically significant Pearson correlations (nominal *p* ≤ 0.05) and their associated r and R² values. *P* values were not adjusted for temporal autocorrelation or multiple testing

At grid-cell scale, September contained broad areas with positive LAI coefficients, whereas NDVI and EVI were mostly weak or negative near Cananéia. In October, negative EVI coefficients occurred in parts of the central and southern domain, with positive values near the Ribeira de Iguape River mouth and in the southern CICS (Fig. 12; Fig. S16 in Online Resource 2).

November and December also differed. In December, moderate positive coefficients covered 33.6% of the domain for NDVI and 34.1% for EVI; strong positive coefficients covered 5.9% and 6.0%, respectively. November LAI was mainly positive, while NDVI and EVI included negative sectors (Fig. 13; Fig. S17 in Online Resource 2). Coefficient and *p*-value maps for January–February, March–April, May–June, and July–August are provided in Figs. S8-S15 in Online Resource 2.

### Three-month associations between air temperature and vegetation indices

When monthly observations were pooled within calendar quarters, AMJ coefficients were positive for NDVI (*r* = 0.29, *p* = 0.04), EVI (*r* = 0.31, *p* = 0.02), and LAI (*r* = 0.32, *p* = 0.02). In OND, EVI and LAI were positive (*r* = 0.44 and 0.48; *p* < 0.005). JAS coefficients were negative for NDVI (*r* =  − 0.31, *p* = 0.03) and EVI (*r* = −0.28, *p* = 0.05). JFM showed a positive LAI association (*r* = 0.61, *R*^2^ = 0.38, *p* = 0.02); NDVI and EVI were not significant (Table 2).

**Table 2:** Pearson correlation coefficient (*r*), coefficient of determination (*R*^2^), and nominal *p* value for area-mean monthly observations pooled within each calendar quarter during 2003–2019

Period	NDVI	EVI	LAI
**JFM**	*r* = − 0.13	*r* = 0.07	***r* = 0.61**
	*R*^2^ = 0.02	*R*^2^ = 0.00	***R***^**2**^** = 0.38**
	*p* = 0.35	*p* = 0.65	***p* = 0.02**
**AMJ**	***r* = 0.29**	***r* = 0.31**	***r* = 0.32**
	***R***^**2**^** = 0.09**	***R***^**2**^** = 0.10**	***R***^**2**^** = 0.10**
	***p* = 0.04**	***p* = 0.02**	***p* = 0.02**
**JAS**	***r* = − 0.31**	***r* = − 0.28**	*r* = 0.13
	***R***^**2**^** = 0.09**	***R***^**2**^** = 0.08**	*R*^2^ = 0.02
	***p* = 0.03**	***p* = 0.05**	*p* = 0.35
**OND**	*r* = 0.19	***r* = 0.44**	***r* = 0.48**
	*R*^2^ = 0.04	***R***^**2**^** = 0.19**	***R***^**2**^** = 0.23**
	*p* = 0.19	***p* < 0.005**	***p* < 0.005**

Values of r and R² were rounded independently from full-precision output; small differences may therefore arise when the displayed r is squared. Values reported as *p* = 0.00 in the source output are shown as *p* 0.005. Bold values indicate statistically significant Pearson correlations (nominal *p* ≤ 0.05) and their associated r and R² values. *P* values were not adjusted for temporal autocorrelation or multiple testing Values of r and R² were rounded independently from full-precision output; small differences may therefore arise when the displayed r is squared. Values reported as *p* = 0.00 in the source output are shown as *p* 0.005. Bold values indicate statistically significant Pearson correlations (nominal *p* ≤ 0.05) and their associated r and R² values. *P* values were not adjusted for temporal autocorrelation or multiple testing

**Fig. 14:**
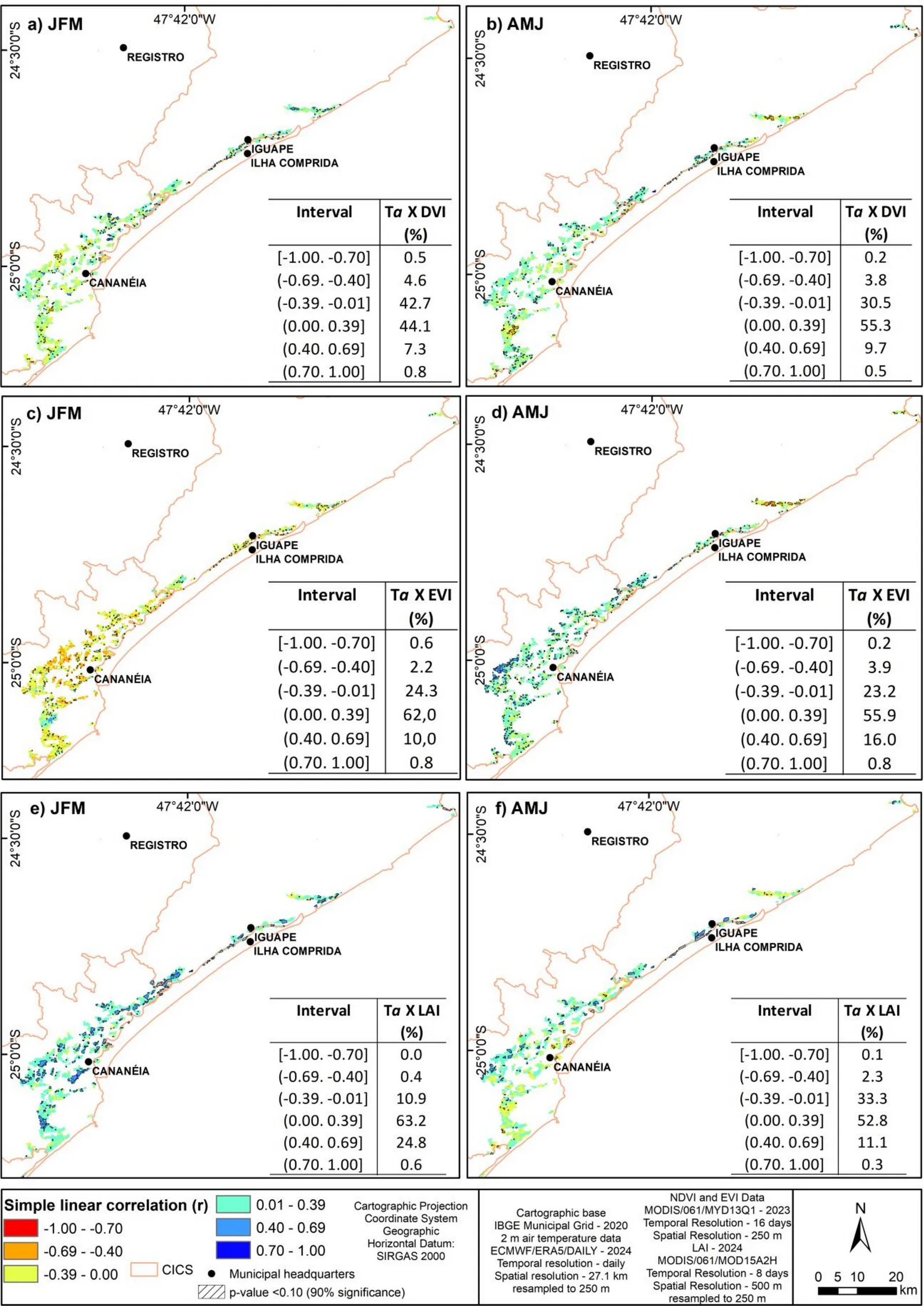
Calendar-quarter grid-cell correlations for JFM and AMJ during 2003–2019. **a**, **b** NDVI. **c**, **d** EVI. **e**, **f** LAI. JFM is in the left column and AMJ in the right column

At grid-cell scale, positive LAI coefficients occupied most of the mapped mangrove in JFM. AMJ was dominated by weak positive coefficients, with negative sectors for individual indices (Fig. 14; Fig. S18 in Online Resource 2).

**Fig. 15:**
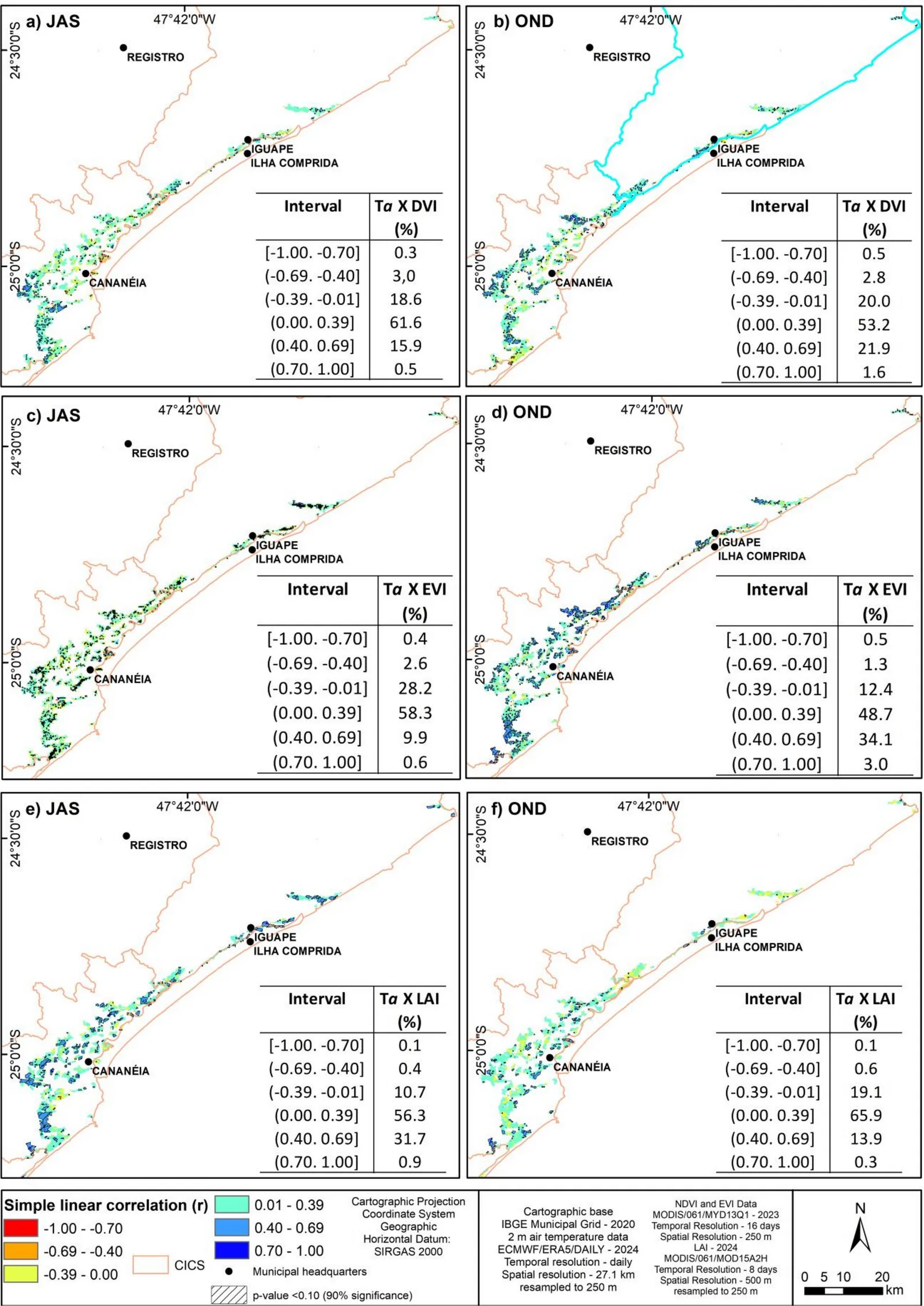
Calendar-quarter grid-cell correlations for JAS and OND during 2003–2019. **a**, **b** NDVI. **c**, **d** EVI. **e**, **f** LAI. JAS is in the left column and OND in the right column

JAS combined negative mangrove-wide NDVI and EVI coefficients with a spatial mixture of weak, moderate, and locally strong relationships. OND had the broadest positive EVI pattern: moderate positive coefficients occupied about 34.1% of the domain and strong positive coefficients 3.0%. NDVI covered a smaller positive area, and LAI was mainly weakly positive (Fig. 15). The associated nominal *p*-value fields are presented in Fig. S19 in Online Resource 2.

## Discussion

### Long-term and intra-annual warming

The annual Sen’s slope of about 0.20 °C decade⁻^1^ is comparable with published trends for mean and extreme temperatures in southeastern Brazil and eastern Paraná (Penereiro & Meschiatti, 2018; Regoto et al., 2021; Terassi et al., 2024). The strongest rates occurred in JAS, particularly September and October, placing the largest warming near the end of the cooler, relatively drier part of the year.

September is a transition month in the CICS. Rainfall begins to recover from its winter minimum while solar radiation, air temperature, and atmospheric water demand increase (Baratto et al., 2024b, 2025; Ferreira & Reboita, 2022). The positive September LAI coefficient coincides with this transition. Because rainfall, radiation, phenology, and freshwater delivery also change during this period, the present data do not identify temperature as an independent driver. Testing these alternatives will require a multivariable analysis.

The area-mean series provides stronger evidence of warming than the grid-cell tests. This contrast is consistent with the short cell-level records, the coarse ERA5 grid, and the absence of corrections for temporal and spatial dependence. Spatial gradients in the interpolated field should therefore be interpreted as regional atmospheric variation rather than canopy or substrate temperature at individual stands.

### Temperature-vegetation associations

The positive annual NDVI and EVI coefficients are consistent with reports that warmer conditions, when they remain within a non-limiting range, can coincide with greater mangrove greenness or canopy development (Khan et al., 2025; Ruan et al., 2022; Simard et al., 2019). At subtropical margins, fewer damaging cold events may also support canopy persistence and range expansion (Cavanaugh et al., 2014; Fazlioglu et al., 2020). Still, the annual *R*^2^ values show that temperature accounts for only part of the interannual variation, and the nonsignificant LAI coefficient indicates that greenness and canopy structure did not follow the same pattern.

The negative JAS coefficients for NDVI and EVI may reflect the timing of temperature relative to freshwater availability. During the cooler, drier interval, relatively warm conditions can coincide with higher atmospheric demand without a comparable increase in rainfall. In JFM and OND, warmer conditions more often occur as rainfall and freshwater inputs recover, which is compatible with the positive EVI and LAI coefficients. These mechanisms remain hypotheses because precipitation, salinity, tidal inundation, radiation, and soil moisture were not included in the models (Alongi, 2015; Ward et al., 2016).

Part of the divergence among coefficients may reflect differences among the satellite products themselves. NDVI and EVI are spectral greenness indices, whereas LAI is a retrieved structural variable that may respond over a different timescale. The products also differ in compositing interval, native spatial resolution, saturation characteristics, quality screening procedures, and retrieval algorithms (Pastor-Guzman et al., 2018; Tran et al., 2022; Zhao et al., 2016). Consequently, disagreement among indices may arise from ecological variation, measurement properties, or both.

No single mechanism adequately explains the negative coefficients in parts of Cananéia and Ilha do Cardoso. They may reflect differences in stand structure, salinity exposure, geomorphology, disturbance history, catchment inputs, or the interpolation of the atmospheric field. Protected-area status limits direct conversion but does not prevent external hydrological or sedimentary changes from reaching the estuary. The June 2019 disturbance near Papagaios Island, which reduced NDVI and LAI, also shows how an episodic event can alter a relationship summarized by quarter-mean temperature (Lima et al., 2023). Site-scale measurements are needed to resolve these contrasts.

### Limitations and research needs

Spatial resolution limits interpretation at the scale of individual mangrove stands. MODIS pixels of 250 and 500 m can mix mangrove with open water, restinga, and other cover along narrow margins. Compositing and quality filters reduce, but do not eliminate, cloud, atmospheric, and mixed-pixel effects. The mangrove-wide and grid-cell components also use vegetation-index products from different satellite platforms. ERA5 2 m air temperature is native to an approximately 0.25° grid. Bicubic interpolation aligns it with MODIS but cannot resolve tidal-channel microclimates, canopy temperature, sharp coastal gradients, or variation within one ERA5 cell.

Spatial resolution limits interpretation at the scale of individual mangrove stands. MODIS pixels of 250 and 500 m can mix mangrove with open water, restinga, and other cover along narrow margins. Compositing and quality filters reduce, but do not eliminate, cloud, atmospheric, and mixed-pixel effects. The mangrove-wide and grid-cell components also use vegetation index products from different satellite platforms. ERA5 2 m air temperature is native to an approximately 0.25° grid, which is coarse relative to the spatial extent and environmental heterogeneity of the Cananéia-Iguape Coastal System. Consequently, the ERA5 field may smooth local land-sea contrasts, estuarine and tidal-channel gradients, and mangrove microclimatic variability. Bicubic interpolation aligns ERA5 with MODIS but changes only the computational grid; it does not create independent climatic information at 250 m resolution. Therefore, the mapped associations should be interpreted as relationships with a regional atmospheric field rather than as measurements of stand-level mangrove, canopy, or substrate temperature.

Thousands of spatial tests were performed without a false discovery rate correction or field significance procedure. Some isolated significant cells may consequently be false positives, while spatial dependence lowers the effective number of independent comparisons. The models also omit lagged responses. A 4-month rainfall lag has been reported previously in the CICS, and delayed thermal or hydrological responses are also possible (Baratto et al., 2024b).

Air temperature was analyzed alone to address a specific gap and to complement earlier CICS studies of precipitation and land surface temperature (Baratto et al., 2024a, b, 2025). Air temperature should therefore be evaluated alongside the hydrological and ecological conditions that regulate mangrove functioning. Priority variables include precipitation, freshwater discharge, salinity, tidal inundation, and radiation, supported by local sensors and permanent field plots. Models that account for lagged responses would provide a firmer basis for attribution.

## Conclusions

ERA5 records sustained atmospheric warming over the CICS mangrove domain from 1979 to 2019. Mean annual temperature rose by about 0.20 °C decade⁻^1^, with the largest quarter-scale and monthly rates in JAS and September. The area-mean trend was consistent, whereas cell level significance was limited and discontinuous.

Annual temperature was positively associated with NDVI and EVI but not with LAI. Within the year, NDVI and EVI were negative in JAS, LAI was positive in JFM, and EVI and LAI were positive in OND. The annual coefficients therefore cannot be interpreted as one uniform canopy response across seasons or across the estuary.

Temperature should remain part of long-term mangrove monitoring in the CICS, but the present analysis does not establish a causal thermal effect. Inference is constrained by the ERA5 grid, MODIS pixel size, temporal dependence, numerous spatial tests, and omission of hydrological covariates. Further work should focus on the September transition and on sites where the mapped patterns recur, combining local air and canopy temperature measurements with rainfall, salinity, tidal inundation, and field observations.

## Supplementary Information

Below is the link to the electronic supplementary material.

ESM 1 (PDF 135 KB)

ESM 2 (PDF 3.88 MB)

## Data Availability

The source datasets used in this study are publicly available through Google Earth Engine and the NASA Land Processes Distributed Active Archive Center. Daily mean ERA5 2 m air temperature was obtained from the ECMWF ERA5 daily collection in Google Earth Engine (ECMWF/ERA5/DAILY) for the 1979–2019 trend analysis and the overlapping 2003–2019 temperature–vegetation analysis. MODIS Collection 6.1 vegetation data for 2003–2019 were obtained from MYD13Q1.061 (Aqua, 16-day NDVI and EVI composites at 250 m resolution; https://doi.org/10.5067/MODIS/MYD13Q1.061) and MOD15A2H.061 (Terra, 8-day LAI/FPAR composites at 500 m resolution; https://doi.org/10.5067/MODIS/MOD15A2H.061). Product identifiers, temporal coverage, scale factors, quality-screening procedures, spatial alignment, and missing-data handling are described in the Materials and methods and documented in Online Resource 1 (ESM_1.pdf). Online Resource 2 (ESM_2.pdf) contains the supplementary spatial trend, correlation, and nominal p-value maps presented in Figs. S1–S19. The processed time series and intermediate map-ready outputs are available from the corresponding author upon reasonable request.

## Code availability

The analysis scripts are available through the public GitHub locations listed in Online Resource 1. That document provides the script inventory, input products, quality-screening rules, temporal aggregation, spatial alignment, complete-case handling, statistical procedures, and expected outputs.
