# Supplementary material for: Assessing mangrove sensitivity to thermal variations through a satellite-based analysis on the southern São Paulo coast, Brazil
Source: Environ Monit Assess. 2026 Oct 9;198(11):1169. doi: 10.1007/s10661-026-16005-6 (PMC13649872; doi:10.1007/s10661-026-16005-6)
Supplement: Supplementary file 1 [file 10661_2026_16005_MOESM1_ESM.pdf]

ONLINE RESOURCE 1

Public analysis scripts and workflow documentation

**Journal**	Environmental Monitoring and Assessment
**Article title**	Assessing mangrove sensitivity to thermal variations through a satellite-based analysis on the southern São Paulo coast, Brazil
**Authors**	Jakeline Baratto; Paulo Miguel de Bodas Terassi; Nádia Gilma Beserra de Lima; Emerson Galvani
**Corresponding author**	Jakeline Baratto
**Affiliation**	Department of Geography, University of São Paulo, 05508-000, São Paulo, São Paulo, Brazil
**E-mail**	jakeline.baratto@usp.br

Purpose

This Online Resource identifies the public scripts and processing decisions used to obtain, quality-screen, aggregate, align, and analyze ERA5 2 m air temperature and MODIS vegetation products for the Cananéia-Iguape Coastal System. It accompanies the methods reported in the manuscript and provides the information required to trace each principal analytical stage.

Data products and access

ERA5 2 m air temperature. ECMWF/ERA5/DAILY in Google Earth Engine; daily mean near-surface air temperature on the native atmospheric grid of approximately 0.25°. Trend period: 1979-2019. Temperature-vegetation period: 2003-2019.

MYD13Q1.061. Aqua MODIS 16-day NDVI/EVI product at 250 m; DOI: https://doi.org/10.5067/MODIS/MYD13Q1.061.

MOD13Q1.061. Terra MODIS 16-day NDVI/EVI product at 250 m; DOI: https://doi.org/10.5067/MODIS/MOD13Q1.061.

MOD15A2H.061. Terra MODIS 8-day LAI/FPAR product at 500 m; DOI: https://doi.org/10.5067/MODIS/MOD15A2H.061.

Mangrove mask. Mapped mangrove extent within the CICS. Mangrove-wide analyses used the mean of all valid mangrove pixels at each time step; grid-cell analyses used co-located complete temperature-vegetation pairs.

Processing sequence

1. Apply the mangrove mask and product-specific quality criteria before temporal aggregation.
2. Apply the MODIS scale factors and retain NDVI, EVI, and LAI as separate variables because their physical meanings, native resolutions, and compositing intervals differ.
3. Aggregate quality-screened observations to monthly values, then derive annual means and the fixed calendar quarters JFM, AMJ, JAS, and OND.
4. Convert ERA5 temperature from kelvin to degrees Celsius before calculating monthly, annual, and calendar-quarter means.
5. For grid-cell comparison, align ERA5 with the 250 m vegetation grid by bicubic interpolation. The operation changes the computational grid but does not add independent climatic information below ERA5 native resolution.
6. Use pairwise complete temperature-vegetation observations; do not replace missing vegetation values by temporal or spatial interpolation.
7. Calculate Pearson r and ordinary least-squares R^2 for the mangrove-wide and grid-cell associations. Use $p \leq 0.05$ as the primary threshold; any $0.05 < p \leq 0.10$ class retained in archived maps is exploratory.
8. Apply the Mann-Kendall test and Sen's median slope only to ERA5 2 m air temperature.
9. Export outputs with explicit variable, period, statistic, unit, and nominal p-value information.

Quality-control and interpretive limits

Pearson correlation describes linear association and does not establish causation. Scatterplots were inspected for conspicuous curvature and influential observations, but the archived workflow did not include formal bivariate-normality testing, prewhitening, autocorrelation correction, robust regression, false-discovery-rate control, or field-significance testing.

The manuscript tables were checked against the source output. R^2 values remain within 0-1. Because r, R^2, and p were calculated at full precision and rounded independently for presentation, the displayed R^2 can differ slightly from the square of the displayed two-decimal r.

The maximum sample sizes are 17 annual pairs, 204 monthly pairs, and 51 pooled monthly pairs per calendar quarter; quality screening and complete-case filtering can reduce the value for an individual coefficient.

ERA5 represents a regional atmospheric field. Neither bicubic alignment nor a fine output grid converts it into a 250 m observation of mangrove canopy or tidal-channel microclimate.

Public script inventory

The following labels correspond to the principal stages of the archived workflow. Full addresses are retained so that the PDF remains usable independently of the manuscript.

S1. MODIS vegetation-index acquisition and aggregation (2003-2019): https://github.com/jakebaratto/P-s-Doutorado-/blob/main/IV's_mensal%20e%20sazonal-new
S2. ERA5 2 m air-temperature acquisition and temporal aggregation (1979-2019): https://github.com/jakebaratto/P-s-Doutorado-/commit/00f258218bec074cb150a55b91d361c19f3f8ce0
S3a. Mangrove-wide annual temperature-vegetation correlations: https://github.com/jakebaratto/P-s-Doutorado-/commit/c9fc93deb89a1a7d6b671b15469a899231b3de54
S3b. Mangrove-wide monthly and calendar-quarter correlations: https://github.com/jakebaratto/P-s-Doutorado-/commit/d49b83d68f0cc0f97128c0ca08f942f61747a4e5
S4a. Annual grid-cell correlations for NDVI and EVI: https://github.com/jakebaratto/P-s-Doutorado-/commit/72f1243f8aa16d557bd7e3fa598f67ef3843937f
S4b. Annual grid-cell correlations for LAI: https://github.com/jakebaratto/P-s-Doutorado-/commit/d9128a2156e7b302abaf93ed701169fa3895a2b2
S4c. Monthly and calendar-quarter grid-cell correlations for NDVI and EVI: https://github.com/jakebaratto/P-s-Doutorado-/blob/main/Correl_espacial_IVs_TAR_mensal_sazonal
S4d. Monthly and calendar-quarter grid-cell correlations for LAI: https://github.com/jakebaratto/P-s-Doutorado-/commit/7fd6c7dcdf6958ce40ad93da261efe262dc100de
S5a. Annual Mann-Kendall and Sen slope analysis for ERA5 temperature: https://github.com/jakebaratto/P-s-Doutorado-/commit/0290fa0125265880d438bd111b0373213a08d579
S5b. Calendar-quarter Mann-Kendall and Sen slope analysis for ERA5 temperature: https://github.com/jakebaratto/P-s-Doutorado-/blob/main/MK_ERA5_temporal_sazonal
S5c. Monthly Mann-Kendall and Sen slope analysis for ERA5 temperature: https://github.com/jakebaratto/P-s-Doutorado-/commit/17ab7182354f21974ac182e4ec4f97ea765b5cda
S6. Annual, monthly, and calendar-quarter spatial trend maps: https://github.com/jakebaratto/P-sDoutorado-/blob/main/MK_ERA5_espacial_mensal_sazonal_anual

Expected outputs

1. ERA5 monthly, annual, and calendar-quarter temperature series and trend summaries;
2. monthly, annual, and calendar-quarter mangrove-wide NDVI, EVI, and LAI series;
3. correlation tables containing r, R^2, nominal p values, and the available complete pairs;
4. grid-cell maps of correlation and nominal p-value classes;
5. Mann-Kendall, Sen's slope, ZMK-class, and nominal p-value maps for ERA5 2 m air temperature.

Availability boundary

The public scripts document the reported workflow. Reproduction also requires the same mangrove mask, product versions, quality-screening choices, analysis periods, and platform services used in the original processing. Processed time series and intermediate map-ready outputs may be requested from the corresponding author.
