# Supplementary material for: Assessing mangrove sensitivity to thermal variations through a satellite-based analysis on the southern São Paulo coast, Brazil
Source: Environ Monit Assess. 2026 Oct 9;198(11):1169. doi: 10.1007/s10661-026-16005-6 (PMC13649872; doi:10.1007/s10661-026-16005-6)
Supplement: Supplementary file 2 [file 10661_2026_16005_MOESM2_ESM.pdf]

ONLINE RESOURCE 2
Supplementary figures

**Journal**	Environmental Monitoring and Assessment
**Article title**	Assessing mangrove sensitivity to thermal variations through a satellite-based analysis on the southern São Paulo coast, Brazil

**Authors**	Jakeline Baratto; Paulo Miguel de Bodas Terassi; Nádia Gilma Beserra de Lima; Emerson Galvani
**Corresponding author**	Jakeline Baratto
**Affiliation**	Department of Geography, University of São Paulo, 05508-000, São Paulo, São Paulo, Brazil
**E-mail**	Jakeline.baratto@usp.br

Figure conventions

Air-temperature trend maps cover 1979-2019, whereas temperature-vegetation association maps cover 2003-2019. JFM, AMJ, JAS, and OND denote fixed calendar quarters (January-March, April-June, July-September, and October-December), not standard meteorological seasons. For the revised interpretation, $p \leq 0.05$ is the primary significance threshold. The $0.05 < p \leq 0.10$ class is retained in the original spatial displays only for traceability and is interpreted as exploratory. Correlation maps show Pearson r classes; p-value maps show nominal significance fields. ERA5 was aligned to the 250 m vegetation grid by bicubic interpolation for computational comparison and should be interpreted as a regional atmospheric field rather than stand-level microclimate.

Cartographic information, satellite products, spatial resolution, data sources, and processing procedures are described in the Methods and Online Resource 1. The captions below identify only the variables, period, panels, units, and statistics necessary to interpret each figure.

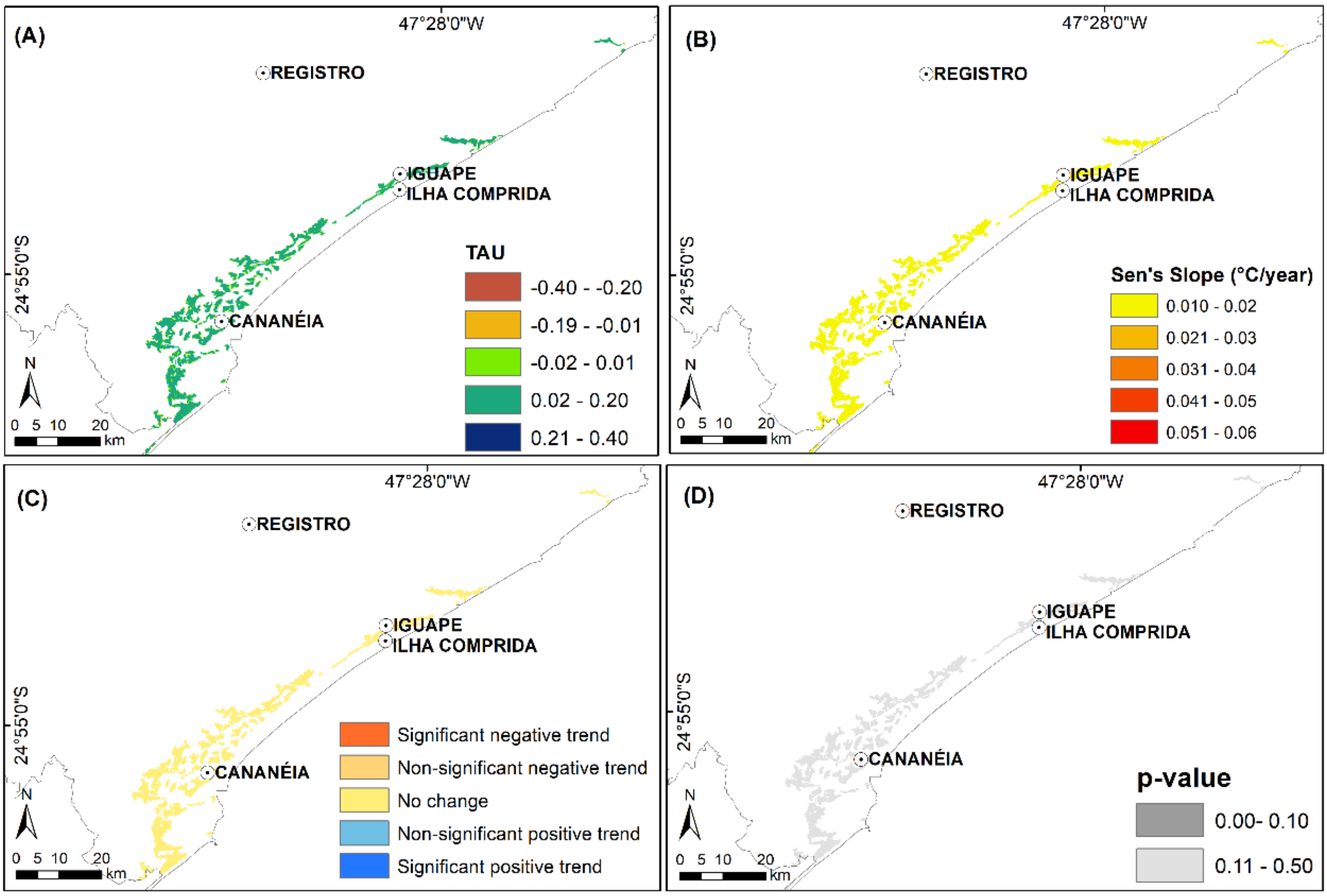

Fig. S1 Spatial trends for annual mean air temperature for the mangrove from 1979 to 2019. Panels show Kendall's tau, Sen's slope (°C year^{-1}), Mann-Kendall trend class, and nominal p value; $p \leq 0.05$ is the primary threshold and $0.05 < p \leq 0.10$ is exploratory.

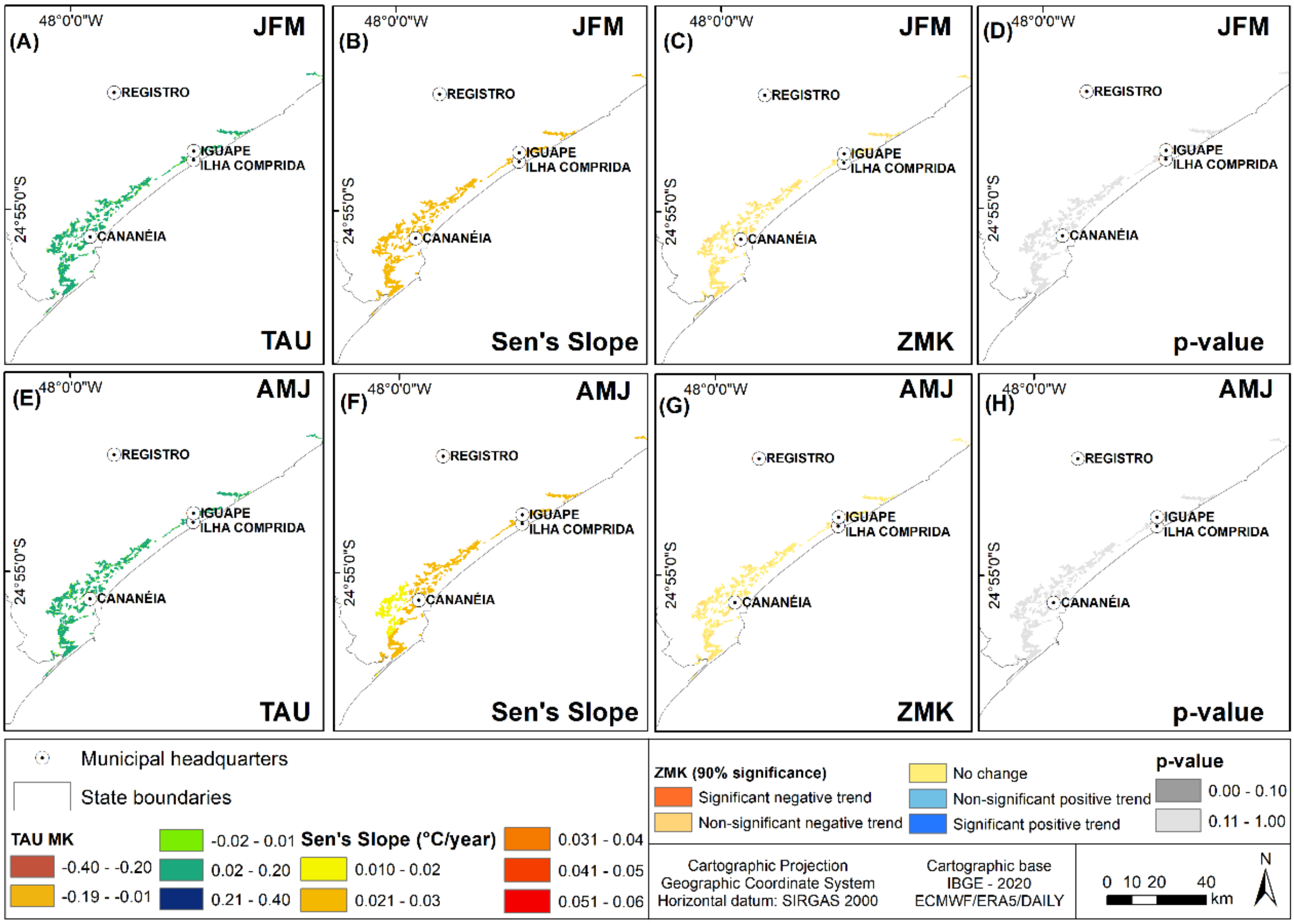

Fig. S2 Spatial trends for calendar-quarter mean air temperature (JFM and AMJ) for the mangrove from 1979 to 2019. The top row shows JFM and the bottom row AMJ; each row presents Kendall's tau, Sen's slope, Mann-Kendall trend class, and nominal p value; $p \leq 0.05$ is the primary threshold and $0.05 < p \leq 0.10$ is exploratory.

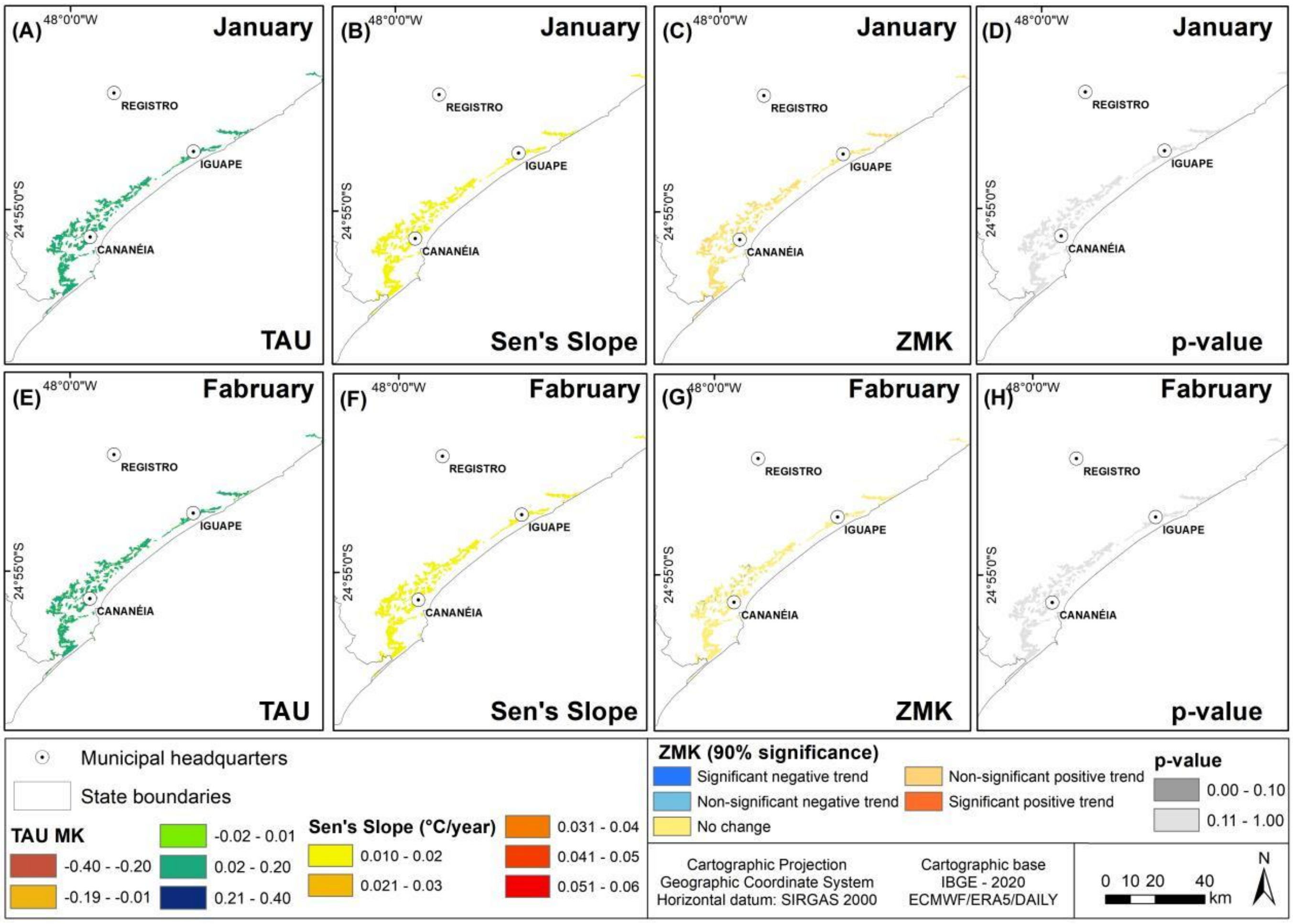

Fig. S3 Spatial trends in mean monthly (January and February) temperature for the mangrove for the period from 1979 to 2019. January is shown in the top row and February in the bottom row; each row presents Kendall's tau, Sen's slope, Mann-Kendall trend class, and nominal p value; $p \leq 0.05$ is the primary threshold and $0.05 < p \leq 0.10$ is exploratory.

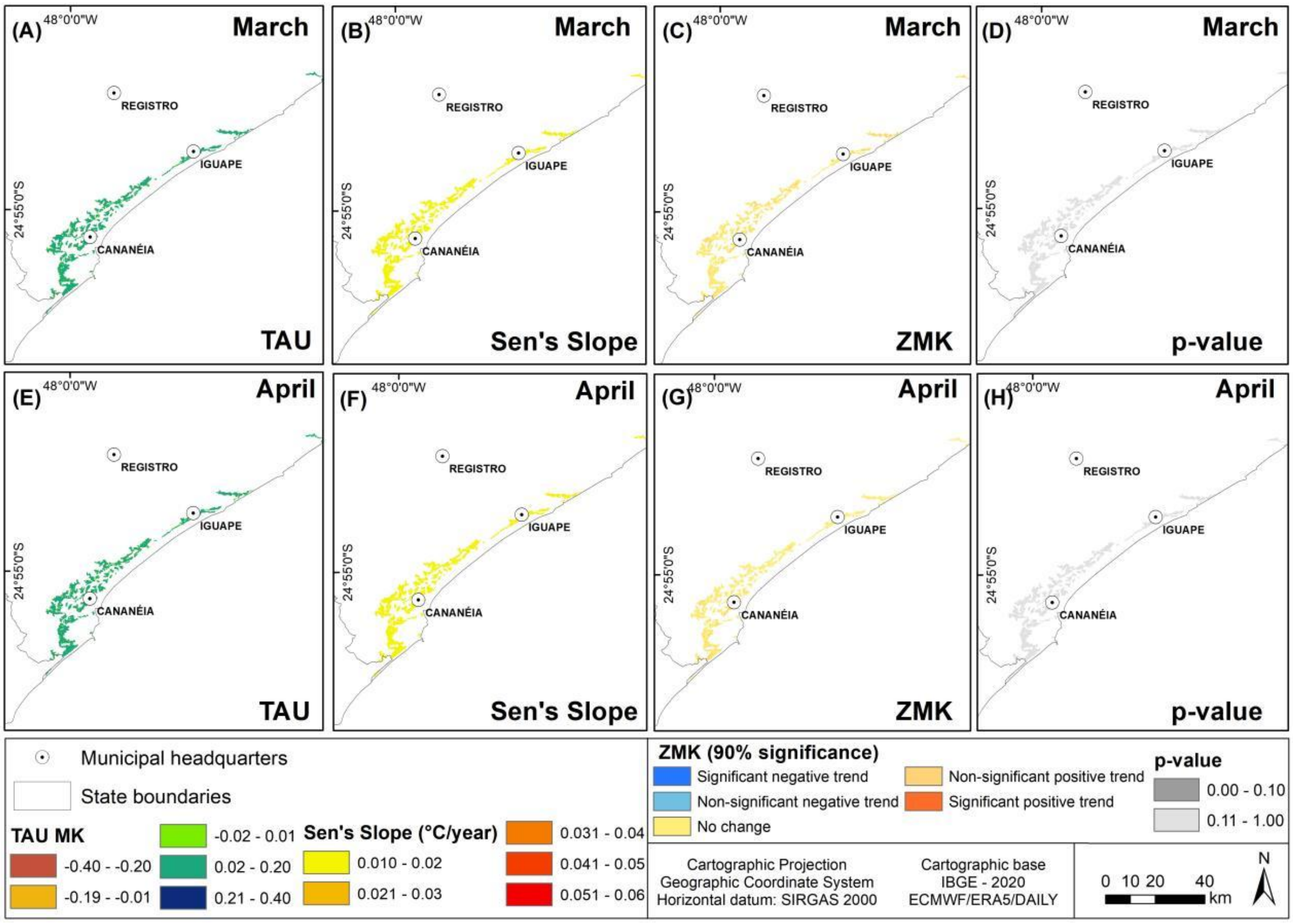

Fig. S4 Spatial trends in mean monthly (March and April) temperature for the mangrove for the period from 1979 to 2019. March is shown in the top row and April in the bottom row; each row presents Kendall's tau, Sen's slope, Mann-Kendall trend class, and nominal p value; $p \leq 0.05$ is the primary threshold and $0.05 < p \leq 0.10$ is exploratory.

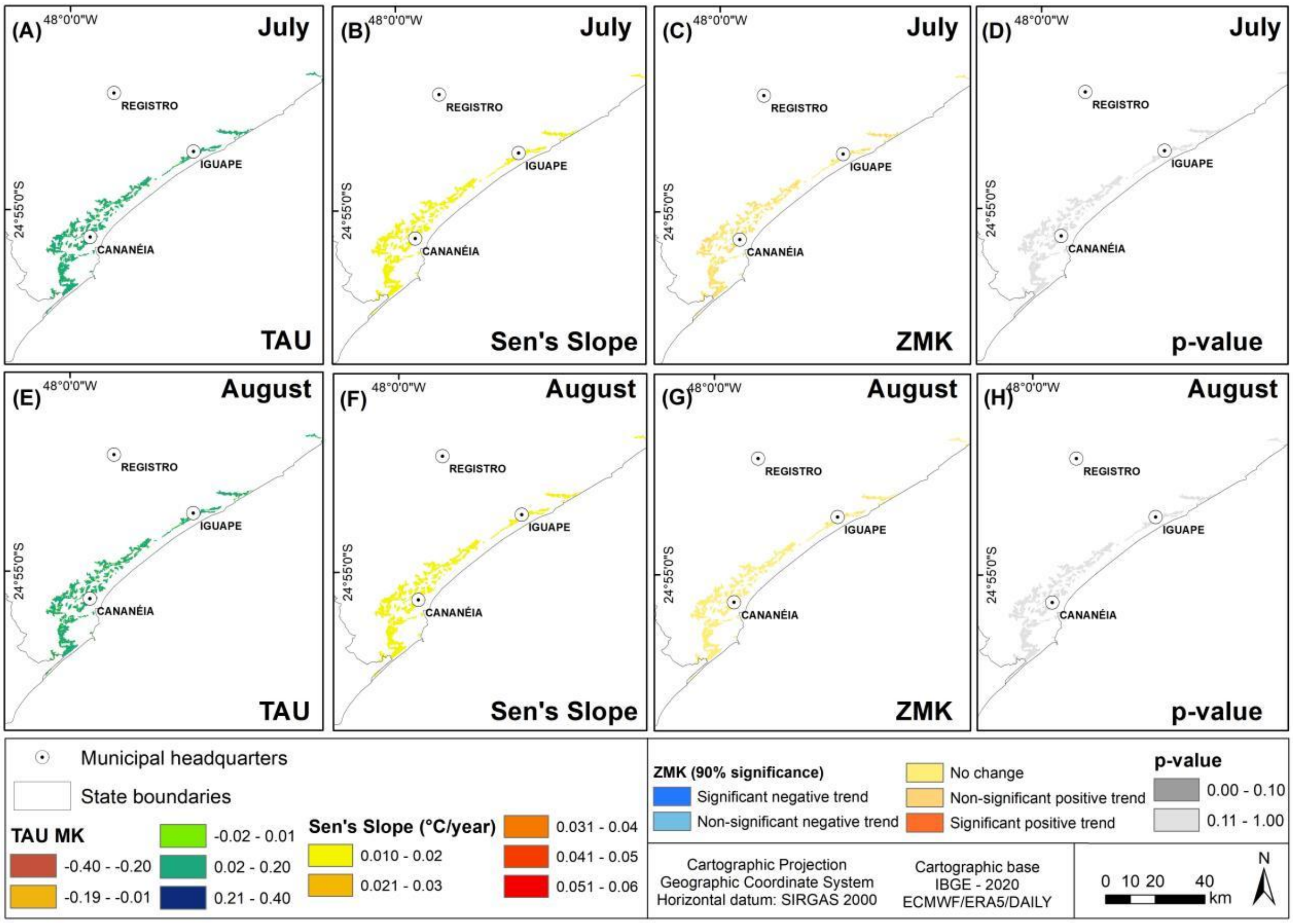

Fig. S5 Spatial trends in mean monthly (July and August) temperature for the mangrove for the period from 1979 to 2019. July is shown in the top row and August in the bottom row; each row presents Kendall's tau, Sen's slope, Mann-Kendall trend class, and nominal p value; p ≤ 0.05 is the primary threshold and 0.05 < p ≤ 0.10 is exploratory.

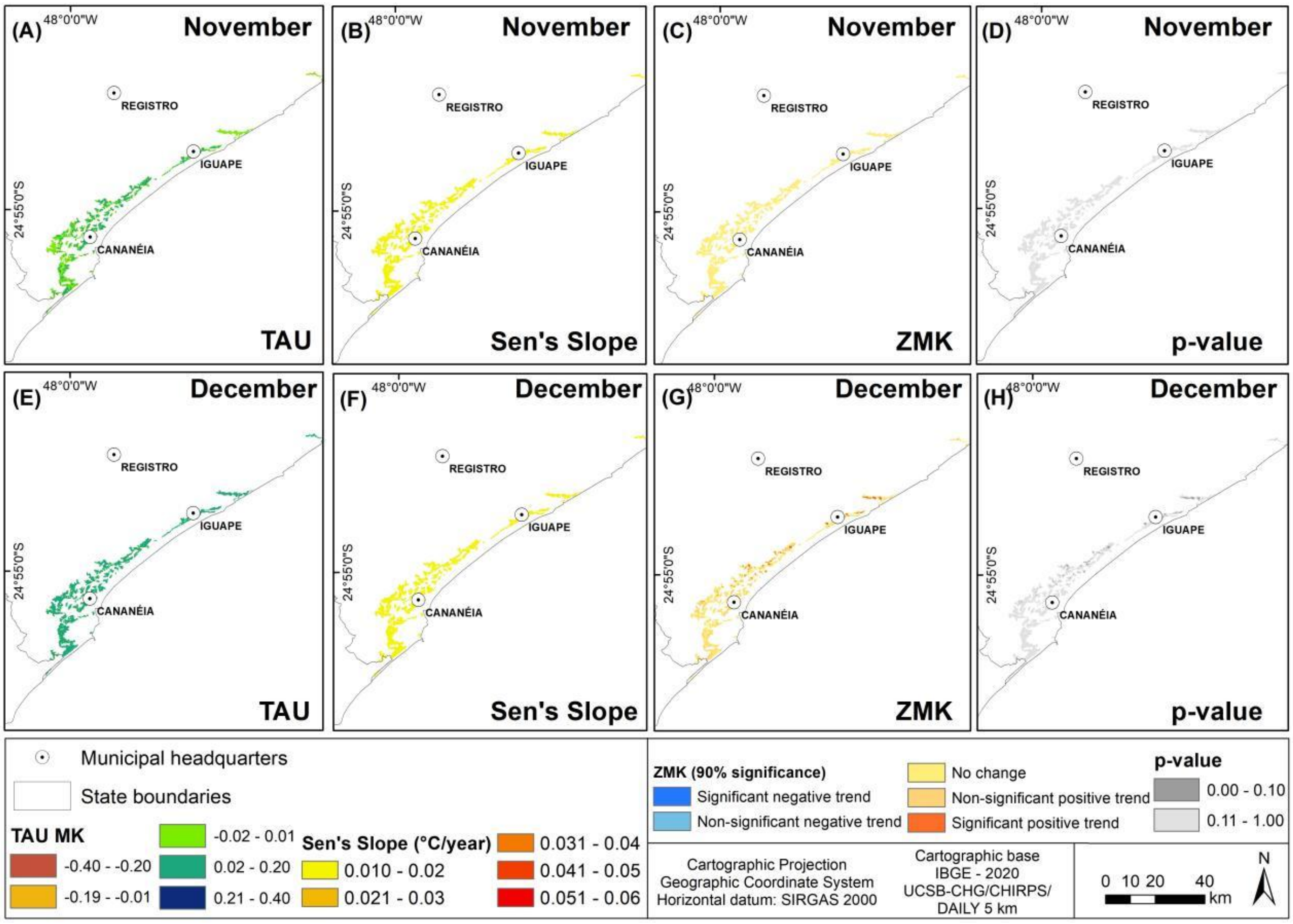

Fig. S6 Spatial trends in mean monthly (November and December) temperature for the mangrove for the period from 1979 to 2019. November is shown in the top row and December in the bottom row; each row presents Kendall's tau, Sen's slope, Mann-Kendall trend class, and nominal p value; $p \leq 0.05$ is the primary threshold and $0.05 < p \leq 0.10$ is exploratory.

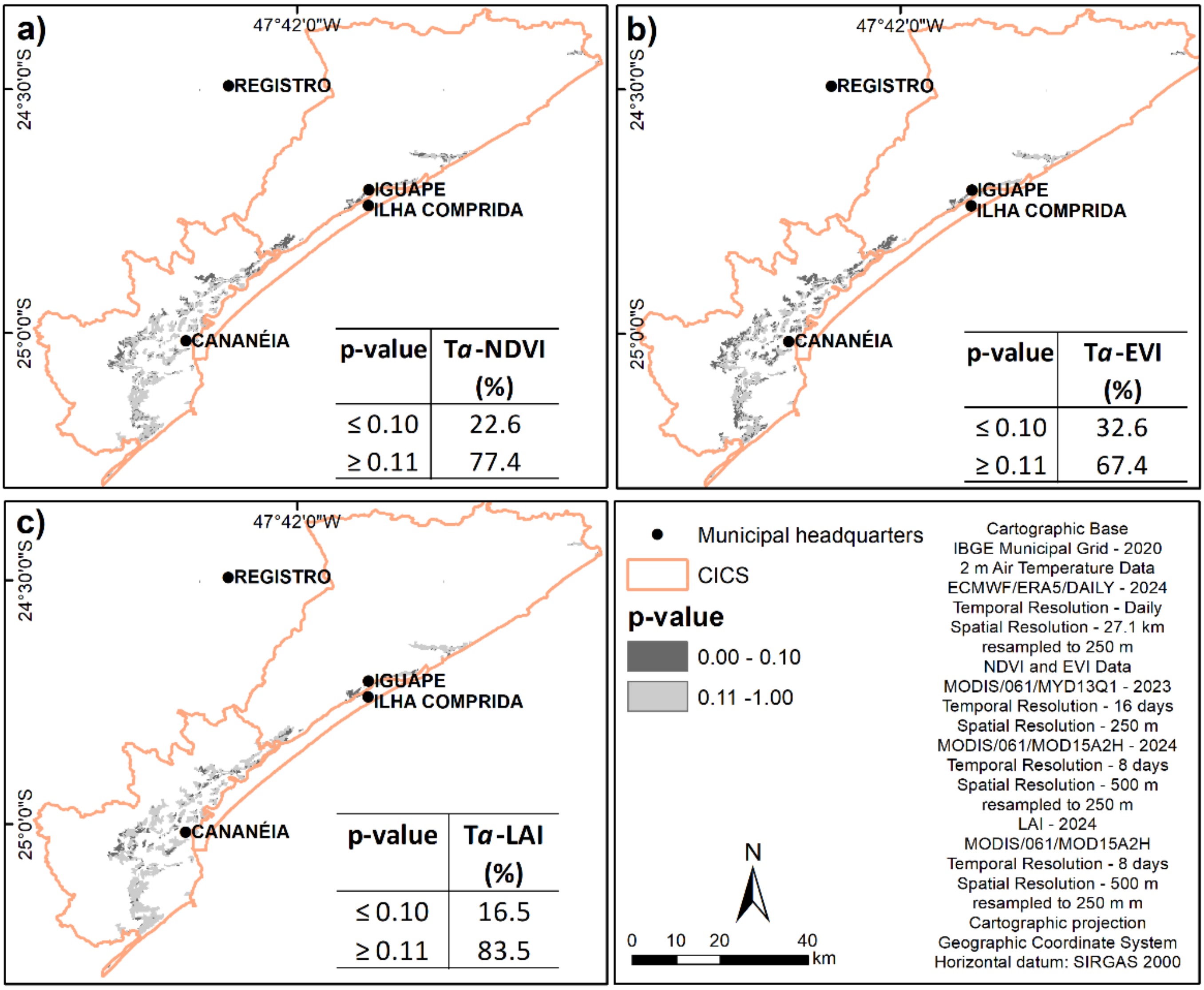

Fig. S7 P-value of the linear correlation between annual air temperature and vegetation indices (2003-2019) of the mangrove swamp of the Cananéia-Iguape Coastal System. Panels (a-c) correspond to NDVI, EVI, and LAI, respectively; $p \leq 0.05$ is the primary threshold and $0.05 < p \leq 0.10$ is exploratory.

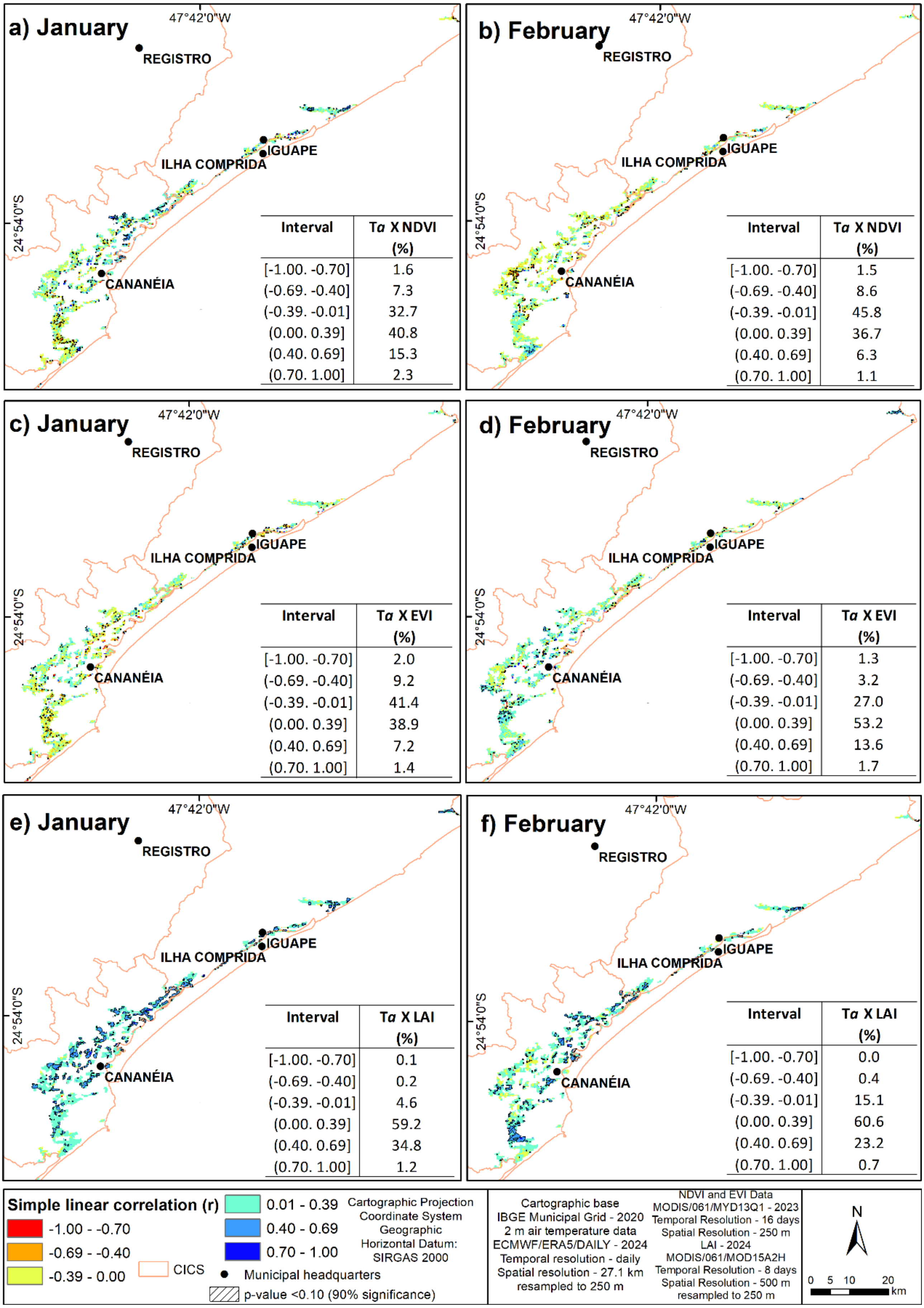

Fig. S8 Linear correlation between air temperature and mangrove vegetation indices (2003-2019) for the January (a, c and e) and February (b, d and f) periods of the Cananéia-Iguape Coastal System. Panels (a, b) show NDVI, (c, d) EVI, and (e, f) LAI; January is in the left column and February in the right column.

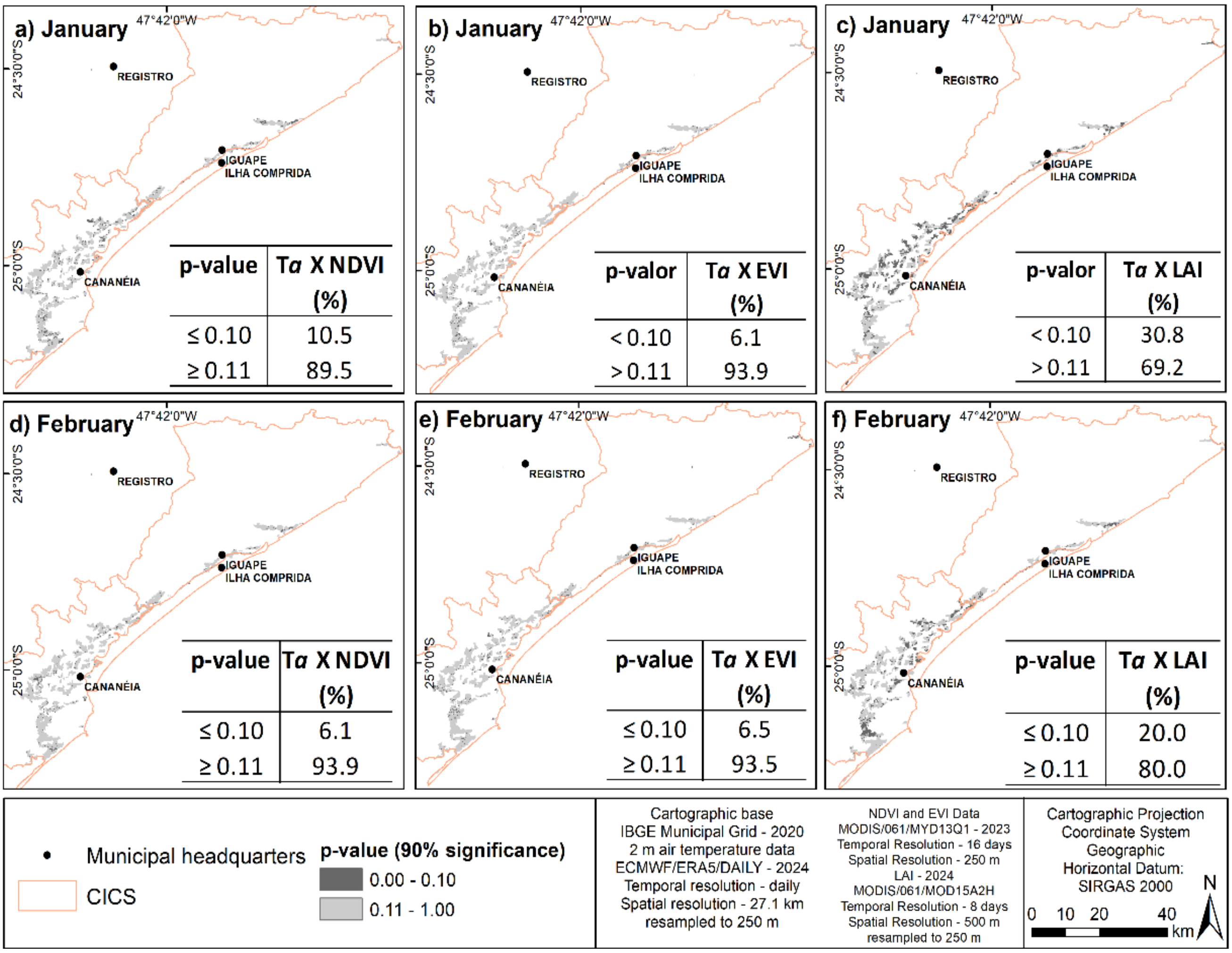

Fig. S9 P-value of the linear correlation between air temperature and mangrove vegetation indices (2003-2019) for the January (a, c and e) and February (b, d and f) periods of the Cananéia-Iguape Coastal System. Panels (a-c) show January and panels (d-f) February; columns correspond to NDVI, EVI, and LAI; $p \leq 0.05$ is the primary threshold and $0.05 < p \leq 0.10$ is exploratory.

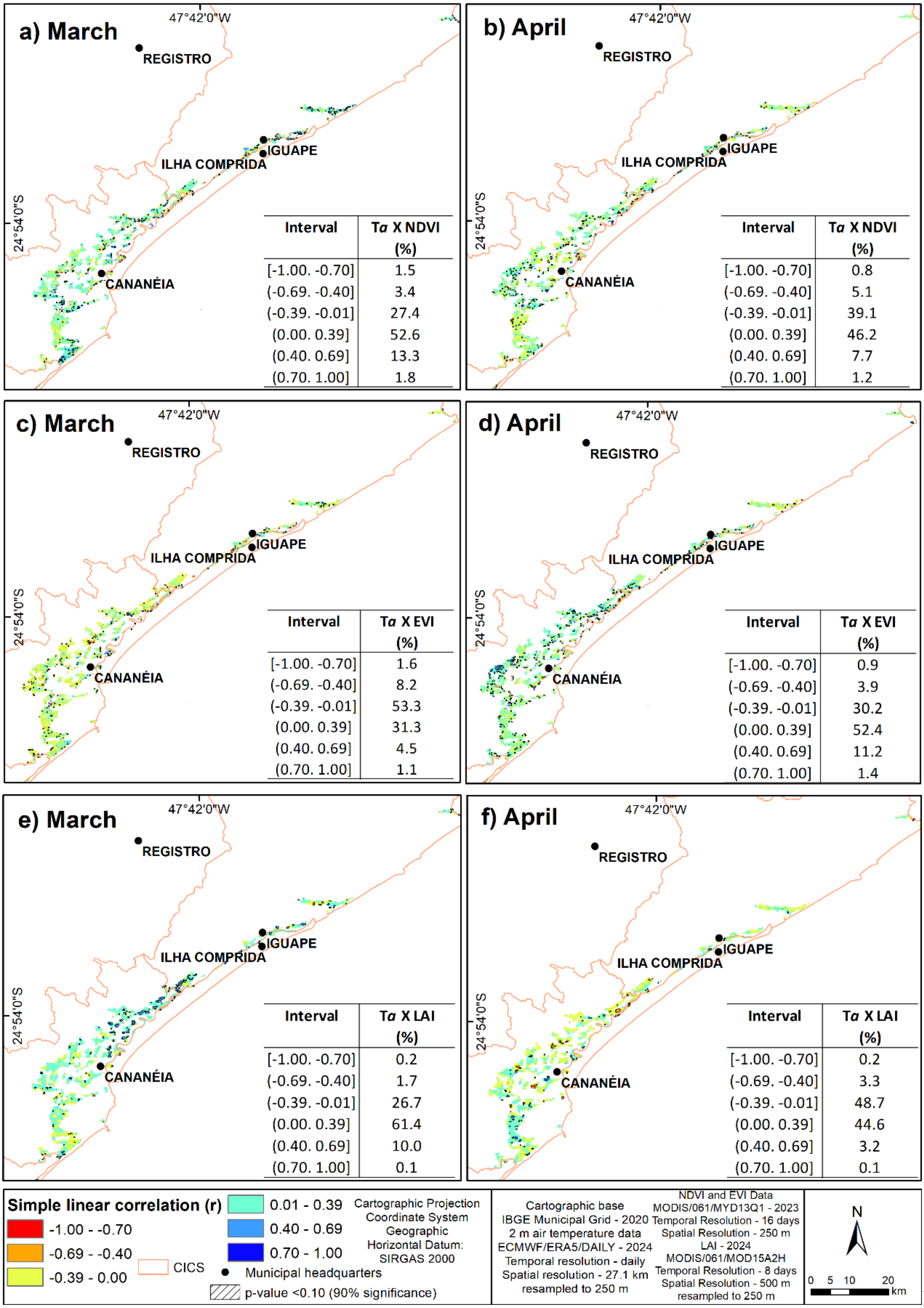

Fig. S10 Linear correlation between air temperature and mangrove vegetation indices (2003-2019) for the March (a, c and e) and April (b, d and f) periods of the Cananéia-Iguape Coastal System. Panels (a, b) show NDVI, (c, d) EVI, and (e, f) LAI; March is in the left column and April in the right column.

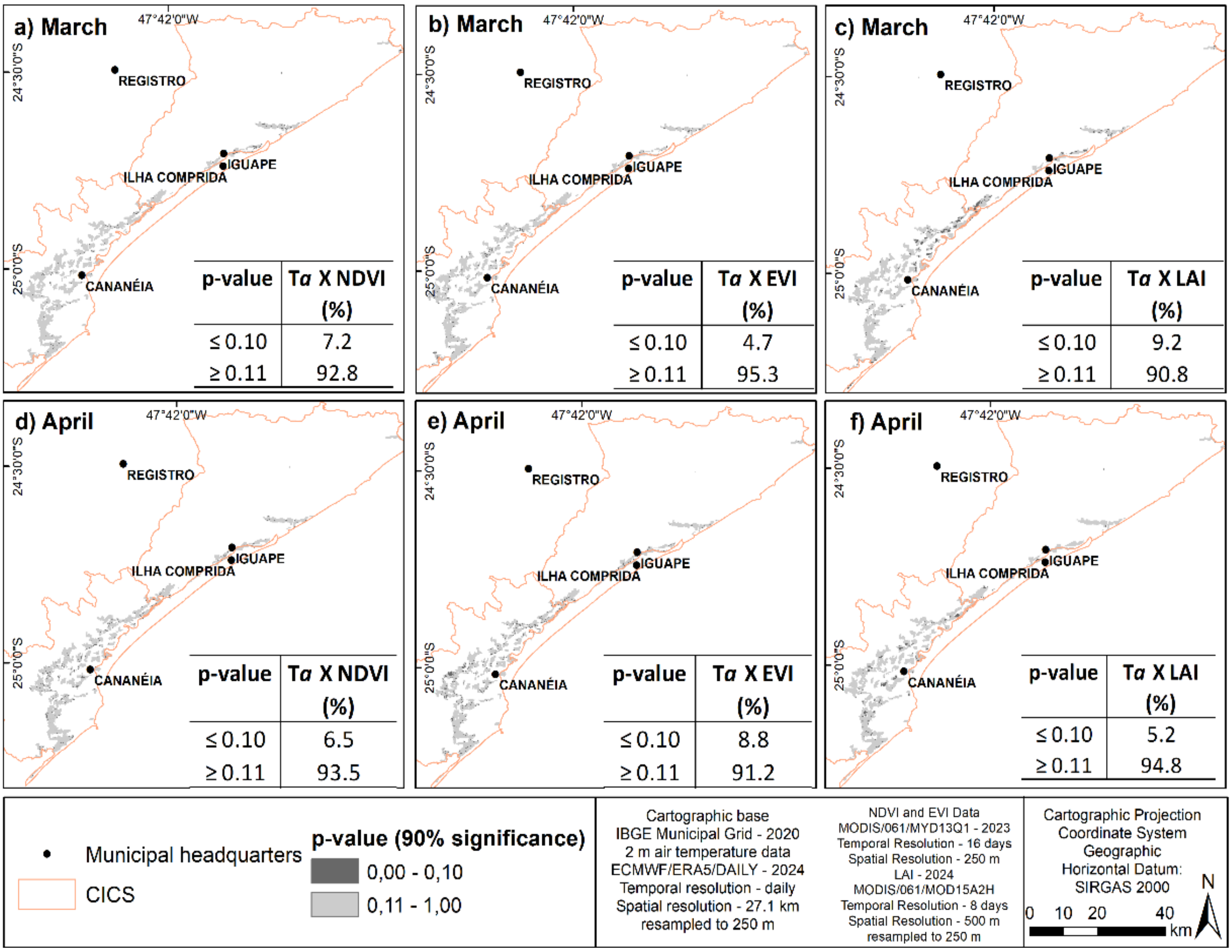

Fig. S11 P-value of the linear correlation between air temperature and mangrove vegetation indices (2003-2019) for the March (a, c and e) and April (b, d and f) periods of the Cananéia-Iguape Coastal System. Panels (a-c) show March and panels (d-f) April; columns correspond to NDVI, EVI, and LAI; p ≤ 0.05 is the primary threshold and 0.05 < p ≤ 0.10 is exploratory.

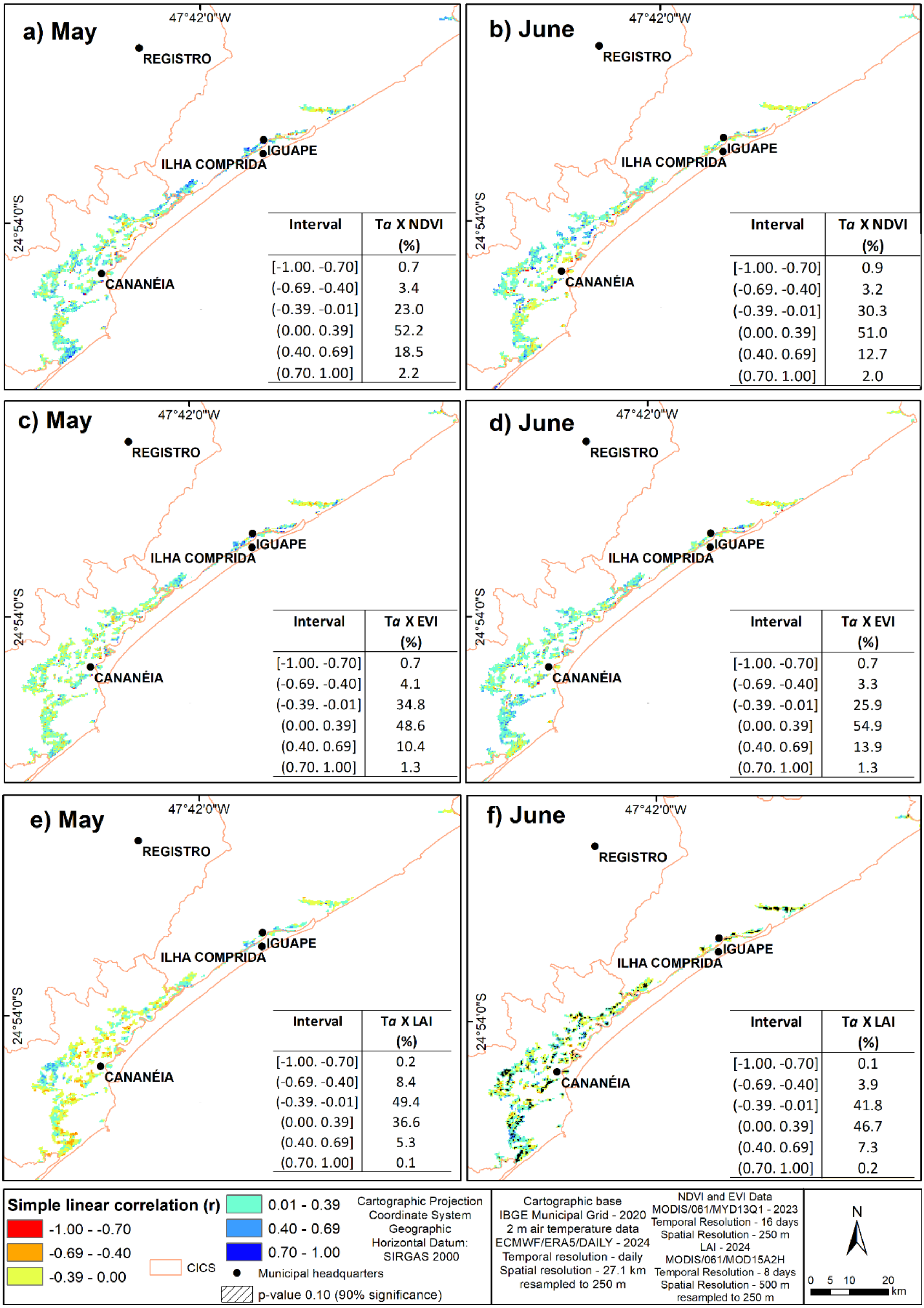

Fig. S12 Linear correlation between air temperature and mangrove vegetation indices (2003-2019) for the May (a, c and e) and June (b, d and f) periods of the Cananéia-Iguape Coastal System. Panels (a, b) show NDVI, (c, d) EVI, and (e, f) LAI; May is in the left column and June in the right column.

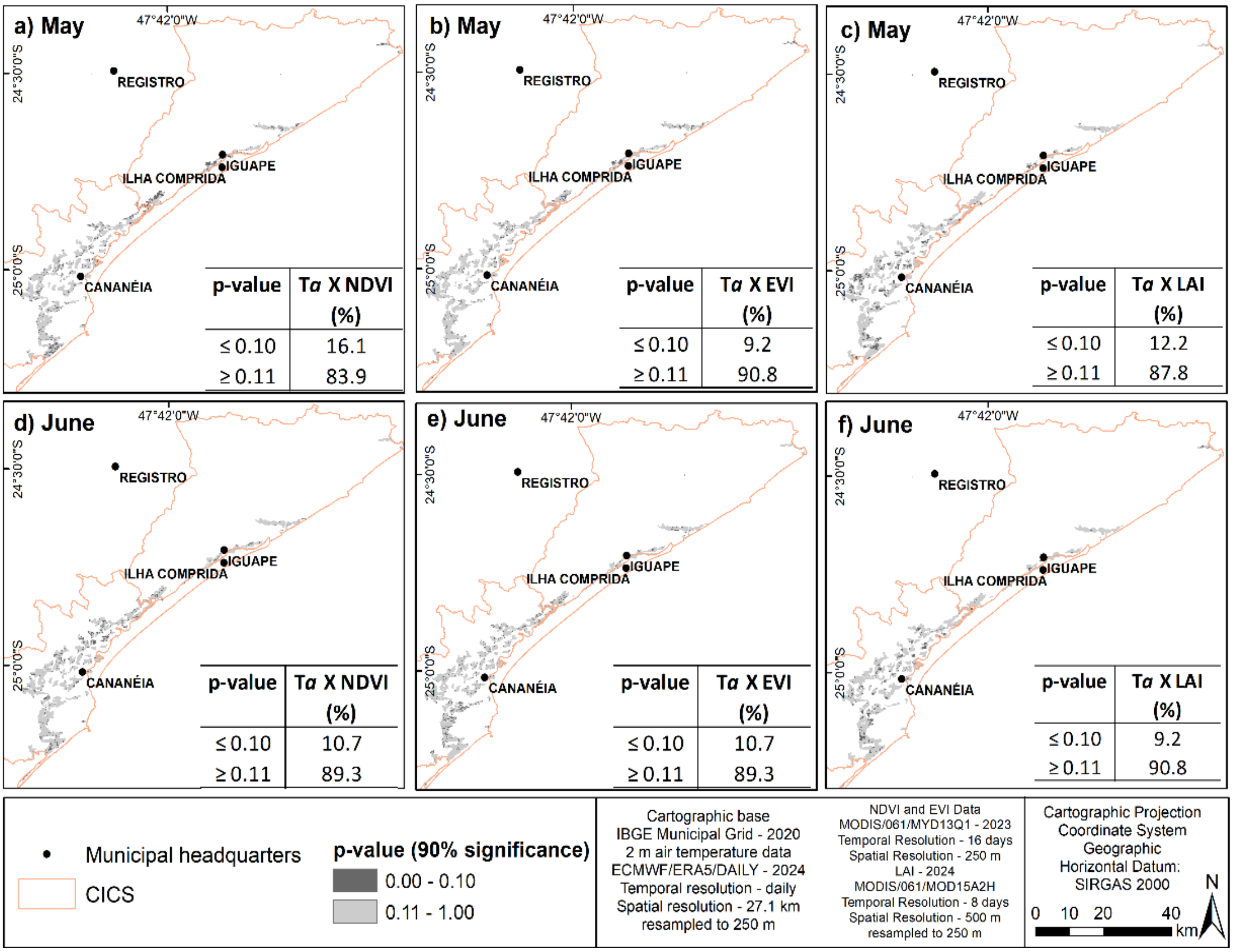

Fig. S13 P-value of the linear correlation between air temperature and mangrove vegetation indices (2003-2019) for the May (a, c and e) and June (b, d and f) periods of the Cananéia-Iguape Coastal System. Panels (a-c) show May and panels (d-f) June; columns correspond to NDVI, EVI, and LAI; $p \leq 0.05$ is the primary threshold and $0.05 < p \leq 0.10$ is exploratory.

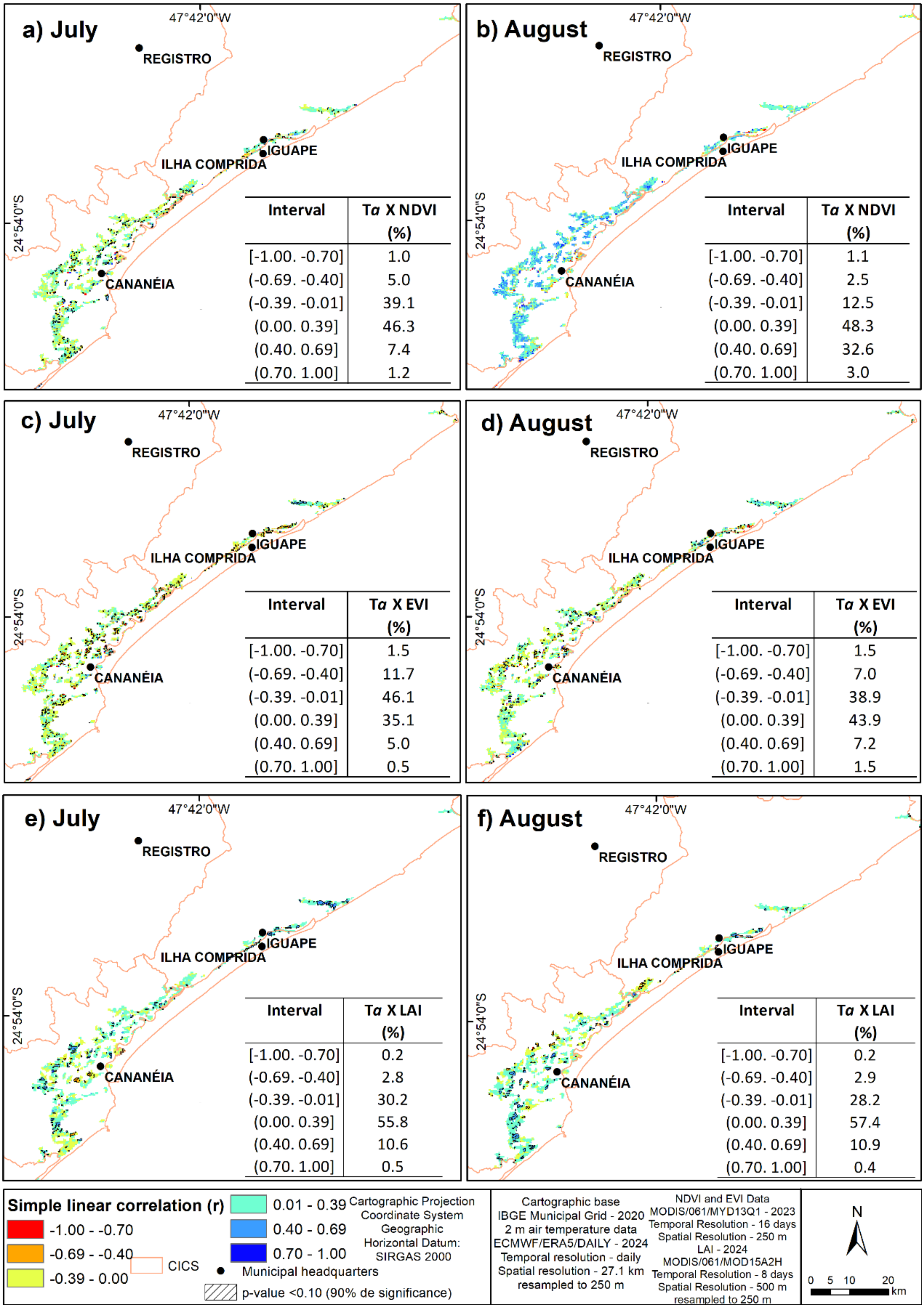

Fig. S14 Linear correlation between air temperature and mangrove vegetation indices (2003-2019) for the July (a, c and e) and August (b, d and f) periods of the Cananéia-Iguape Coastal System. Panels (a, b) show NDVI, (c, d) EVI, and (e, f) LAI; July is in the left column and August in the right column.

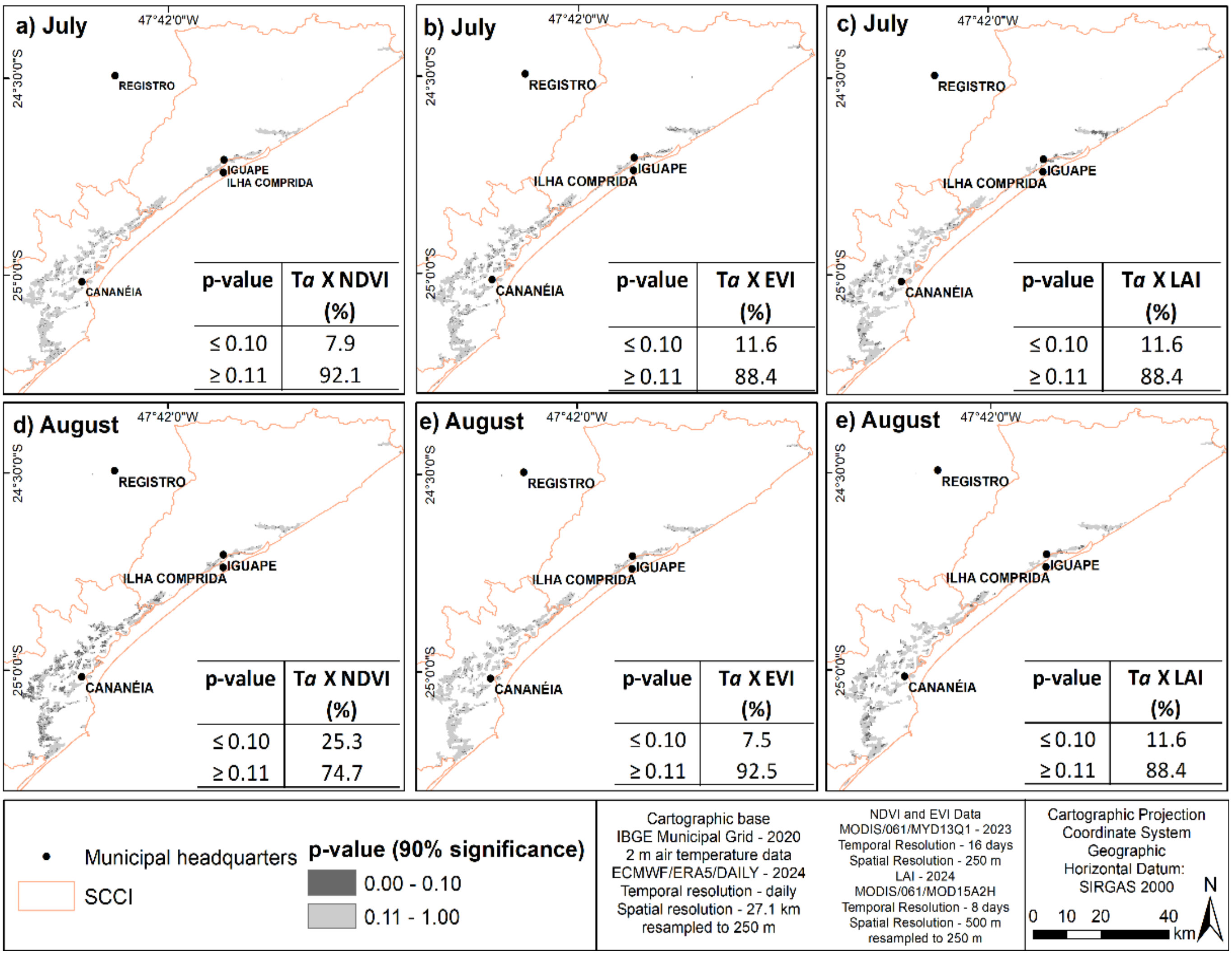

Fig. S15 P-value of the linear correlation between air temperature and mangrove vegetation indices (2003-2019) for the July (a, c and e) and August (b, d and f) periods of the Cananéia-Iguape Coastal System. Panels (a-c) show July and panels (d-f) August; columns correspond to NDVI, EVI, and LAI; $p \leq 0.05$ is the primary threshold and $0.05 < p \leq 0.10$ is exploratory.

Fig. S16 P-value of the linear correlation between air temperature and mangrove vegetation indices (2003-2019) for the September (a, c and e) and October (b, d and f) periods of the Cananéia-Iguape Coastal System. Panels (a-c) show September and panels (d-f) October; columns correspond to NDVI, EVI, and LAI; p ≤ 0.05 is the primary threshold and 0.05 < p ≤ 0.10 is exploratory.

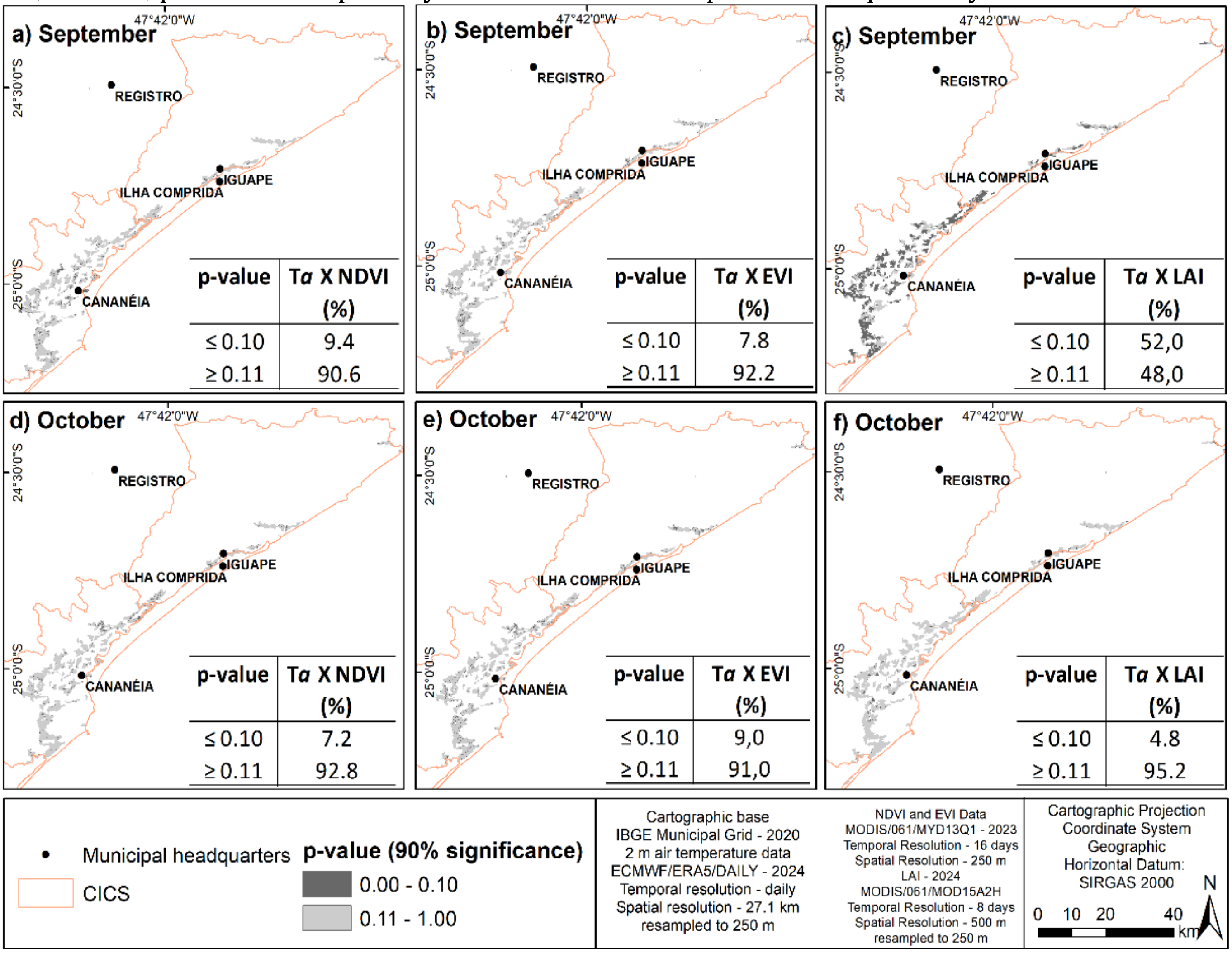

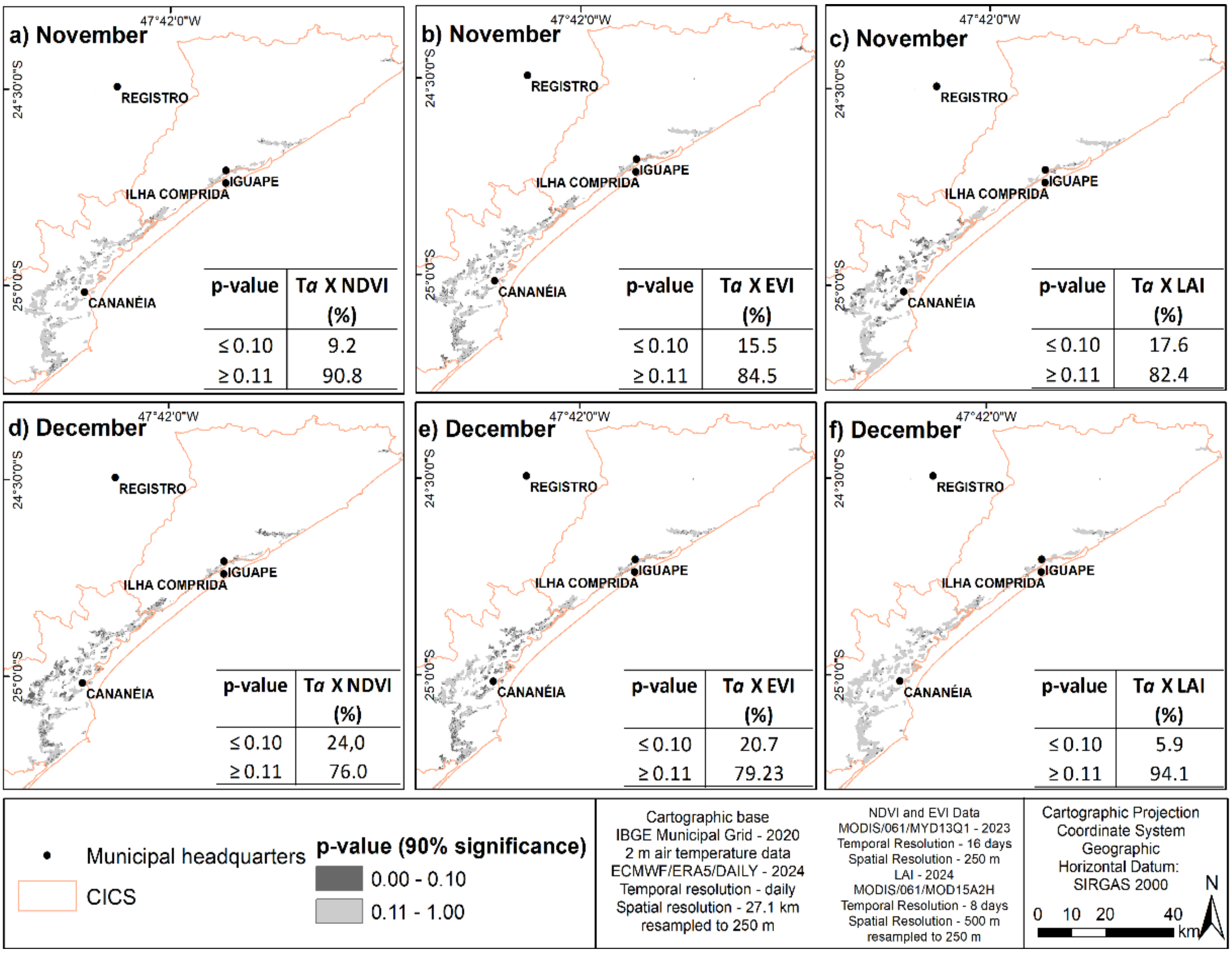

Fig. S17 P-value of the linear correlation between air temperature and mangrove vegetation indices (2003-2019) for the November (a, c and e) and December (b, d and f) periods of the Cananéia-Iguape Coastal System. Panels (a-c) show November and panels (d-f) December; columns correspond to NDVI, EVI, and LAI; $p \leq 0.05$ is the primary threshold and $0.05 < p \leq 0.10$ is exploratory.

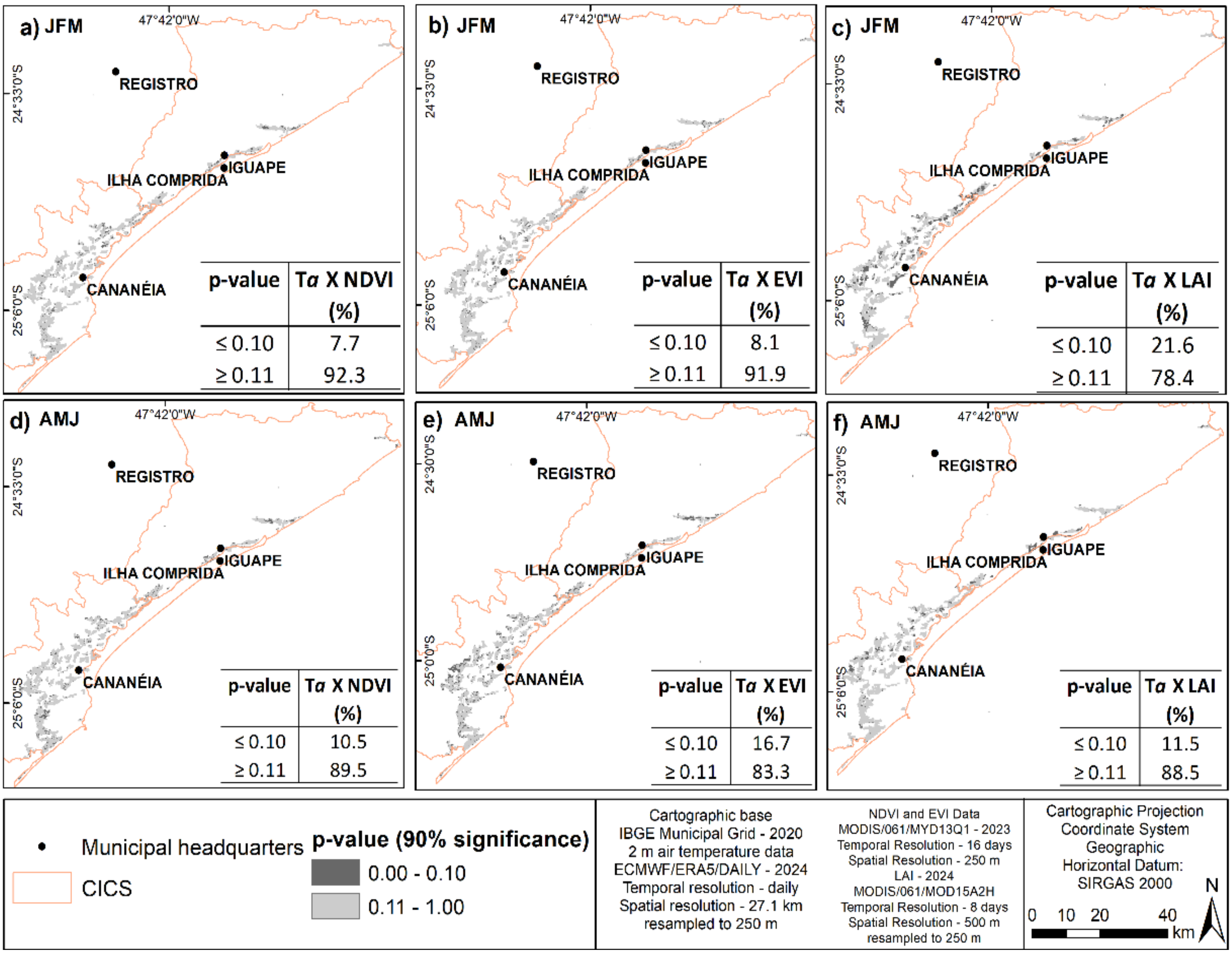

Fig. S18 P-value of the linear correlation between air temperature and mangrove vegetation indices (2003-2019) for the JFM (a, b and c) and AMJ (d, e and f) periods of the Cananéia-Iguape Coastal System. Panels (a-c) show JFM and panels (d-f) AMJ; columns correspond to NDVI, EVI, and LAI; $p \leq 0.05$ is the primary threshold and $0.05 < p \leq 0.10$ is exploratory.

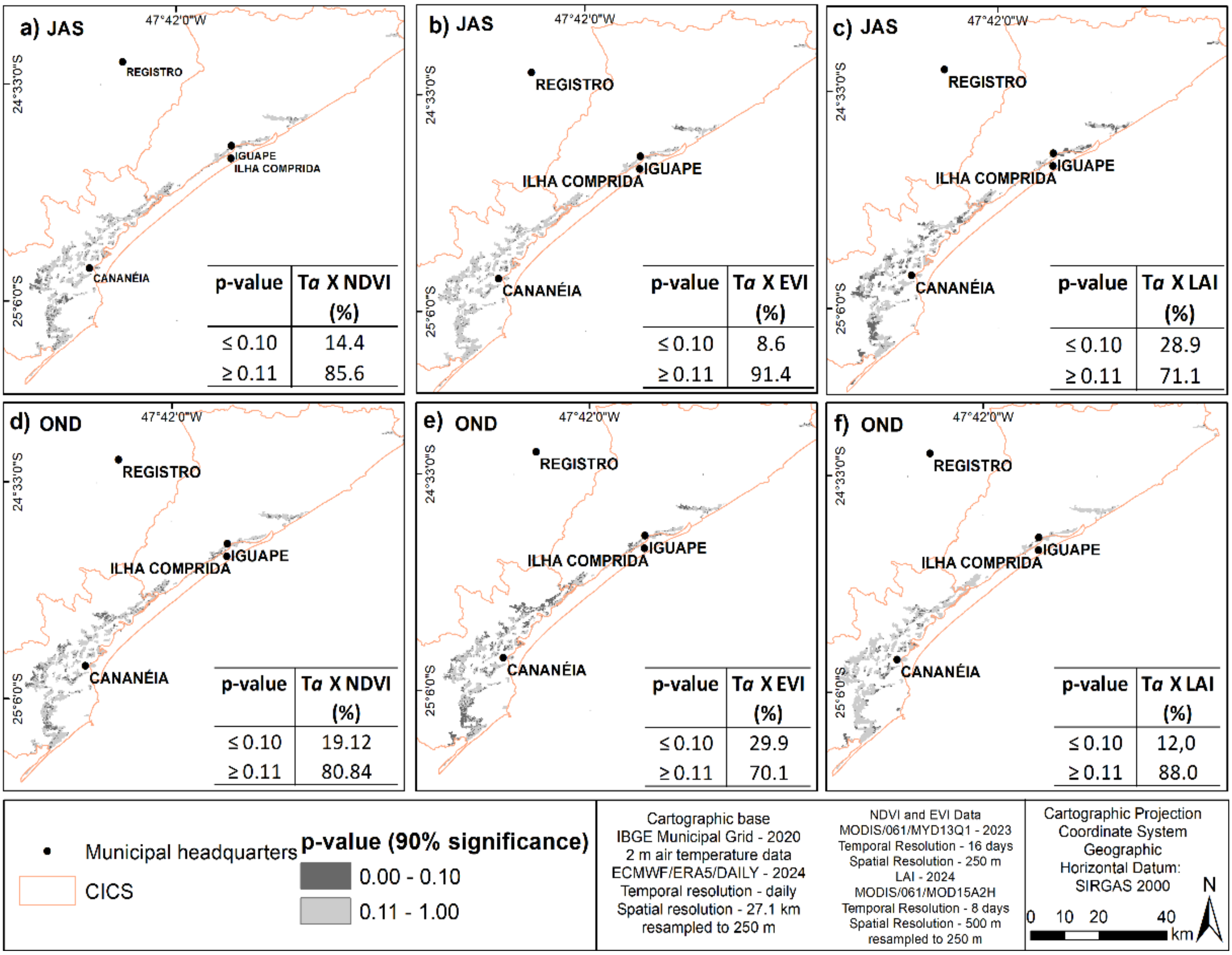

Fig. S19 P-value of the linear correlation between air temperature and mangrove vegetation indices (2003-2019) for the JAS (a, b and c) and OND (d, e and f) periods of the Cananéia-Iguape Coastal System. Panels (a-c) show JAS and panels (d-f) OND; columns correspond to NDVI, EVI, and LAI; $p \leq 0.05$ is the primary threshold and $0.05 < p \leq 0.10$ is exploratory.
